# Extreme temperatures, recent warming and seasonal influenza-linking human exposures to respiratory health in southern Germany

**DOI:** 10.1007/s00420-025-02179-y

**Published:** 2025-11-22

**Authors:** Matteo Boser, Daria Luschkova, Monika Seemann, EXTREME Study Group, Monika Seemann, Gertrud Hammel, Anna Lang, Julia Sander, Iñaki Soto Rey, Markus Wehler, Katharina Zeiser, Claudia Traidl-Hoffmann, Maria P. Plaza

**Affiliations:** 1 Institute of Environmental Medicine and Integrative Health - Environmental Medicine, Faculty of Medicine, University of Augsburg and University Hospital of Augsburg, Augsburg, Germany; 2 Institute of Environmental Medicine, Environmental Health Center, Helmholtz Zentrum München, Neuherberg, Germany; 3 Department of Dermatology and Allergy, University Hospital Augsburg, Augsburg, Germany; 4 Christine Kühne Center for Allergy Research and Education (CK-CARE), Davos, Switzerland

**Keywords:** Extreme temperature, Respiratory health, Time series analysis, Vulnerable populations, Climate change, Health care utilization

## Abstract

**Purpose:**

In the context of climate change, extreme ambient temperatures pose a major threat to human health. This study aims to provide detailed insights into how extreme temperatures and potential confounders affect respiratory morbidity.

**Methods:**

We employed a 14-year time series analysis (2006–2019) in southern Germany, applying penalized distributed lag non-linear models to estimate exposure–response relationships between extreme temperatures and respiratory health outcomes, considering emergency outpatient treatments and hospital admissions at the emergency department of the University Hospital Augsburg. We thereby explored the roles of relative humidity and seasonal influenza as potential confounders and vulnerabilities related to age and gender.

**Results:**

We found significantly elevated relative risks (RRs) for the short-term cumulative effect (Lag: 0–3 days) of extreme heat and the long-term cumulative effect (Lag: 0–21 days) of extreme cold on outpatient treatments and hospital admissions. Seasonal influenza was identified as a significant confounder, with attributable fractions comparable to those of cold temperatures. A sub-period analysis (2006–2012 and 2013–2019) revealed a correlation between the recent rise in temperature and the strong increase in the estimated short-term cumulative effect of extreme heat on hospital admissions (2006–2012, RR: 1.08 (95%CI [0.91, 1.27]) vs. 2013–2019, RR: 1.32 (95%CI [1.15, 1.51]).

**Conclusion:**

Our study demonstrated that extreme temperatures significantly affect respiratory morbidity, with notable influences from seasonal influenza. Sub-period analysis indicated that rising temperatures are already translating into measurable effects on respiratory health, foreshadowing the potentially devastating impacts of global warming on human health. Our results thereby provide highly relevant insights to support targeted public healthcare interventions.

**Supplementary Information:**

The online version contains supplementary material available at 10.1007/s00420-025-02179-y.

## Introduction

Extreme ambient temperatures play a critical role in the context of the adverse impacts of climate change on human health (Agache et al. 2022; Traidl-Hoffmann et al. 2023). Among the various health issues linked to temperature extremes, the respiratory system is particularly sensitive to environmental conditions (D’Amato et al. 2014; Götschke et al. 2017; Romanello et al. 2024). Recent research has established significant associations between extreme ambient temperatures and respiratory-related morbidity and mortality (Achebak et al. 2024; Andersen et al. 2023; Iñiguez et al. 2021; Martínez-Solanas and Basagaña, 2019; Wen et al. 2024). Both extreme heat and cold have been demonstrated to exacerbate respiratory symptoms, trigger acute episodes, and increase rates of respiratory-related health care utilization, thereby underpinning the role of temperature as a significant environmental stressor. These findings highlight not only the vulnerability of our societies to climate-related health impacts, but also hint at the additional strain on the already severely burdened healthcare systems (Agache et al. 2022).

Extreme heat typically refers to temperatures that substantially exceed the historical averages for a particular location and period of time. Extreme heat events are often associated with severe health risks, particularly among vulnerable populations (Ebi et al. 2021; Wen et al. 2024; Winklmayr et al. 2022). In recent years, global temperatures have been consistently above average, with regions such as southeastern Europe, North Africa and the Middle East experiencing frequent and unusual severe heatwaves (Copernicus 2025). Even in Germany, a country with a predominantly temperate climate, heat-related morbidity and mortality pose a growing threat to the aging population and the public health care system (Frasch et al. 2025; Karlsson and Ziebarth 2018; Winklmayr et al. 2022). Changes in atmospheric circulation could play a major role in understanding why extreme heat in Western Europe has increased even more than expected, with several unprecedented heat waves over the last 20 years (Vautard et al. 2023). In regions unaccustomed to extreme heat, the population is particularly vulnerable when such events occur (Clarke et al. 2022; Thompson et al. 2023). Among the vulnerable populations are individuals with pre-existing respiratory conditions, resulting in a notable rise in exacerbations of chronic conditions, including chronic obstructive pulmonary disease (COPD) and asthma (Bernstein and Rice 2013). Urban environments with heightened air pollution (Berger et al. 2020) and the urban heat island effect (Piracha and Chaudhary 2022) can further exacerbate the impact of heat. Air pollutants such as ground-level ozone and fine particulate matter, both of which are known respiratory irritants (Ebi and McGregor 2008; Li et al. 2023), tend to increase with rising temperatures, especially during stagnant weather conditions (Jacob and Winner 2009).

Extreme cold temperatures also exert a strong effect on the respiratory system. Exposure to cold air can cause bronchoconstriction, the narrowing of the airways, which produces symptoms such as wheezing and breathlessness (Koskela 2007). The response is particularly severe in people with asthma, for whom cold air often induces acute exacerbations (D’Amato et al. 2018). There is evidence that in temperate climates, cold temperatures and low humidity, typical of the winter season, can facilitate the transmission of respiratory viruses (Peci et al. 2019). Furthermore, cold-induced vasoconstriction may compromise the immune response in the respiratory tract, making people more likely to become infected and worsening symptoms (Mourtzoukou and Falagas 2007). In particular during winter, seasonal influenza viruses can exert a substantial impact on mortality (Lytras et al. 2019; Schindler et al. 2024) and morbidity (Schindler et al. 2024). Yet the impacts of cold exposure are not constrained to the respiratory system, but can also cause raised blood pressure and worsening cardiovascular conditions (Phung et al. 2016).

In times of global warming, rising temperatures and an increased frequency of extreme weather events are expected to worsen respiratory health impacts (Covert et al. 2023; D’Amato et al. 2014; Ebi et al. 2021). Effectively mitigating these risks requires a clear understanding of how temperature extremes affect respiratory health, which can vary depending on regional climatic conditions, population vulnerabilities and access to medical care (Åström et al. 2013; Yin et al. 2023). Socioeconomic status is also a critical factor, as both absolute and relative poverty are associated with an increased susceptibility to the adverse health effects of extreme temperatures (Covert et al. 2023). Although various protective solutions like patient education and heat monitoring have been proposed to reduce these risks, there are some uncertainties regarding the effectiveness of the recommended measures (Hannemann et al. 2024; Johar et al. 2025; Li et al. 2015).

In recent years, a large number of studies have investigated the influence of extreme temperature on mortality, ranging from all-cause to cause-specific assessments (Gasparrini et al. 2015; Huber et al. 2020; Masselot et al. 2023; Vicedo-Cabrera et al. 2021). To accurately assess the impact of extreme temperatures on both individuals and public health care systems, we deem it necessary to go beyond the usual mortality assessment and additionally analyze cause and treatment setting specific impacts on morbidity. This is crucial for reliable and differentiated assessments of the additional economic and social burden expected due to climate change. Further a growing number of studies tend to take a multi-country or multi-city approach (Gasparrini et al. 2015; Masselot et al. 2023; Vicedo-Cabrera et al. 2021), pooling results from several locations for enhancing statistical power. While these studies provide robust evidence at the macro level, they can mask important local variations, since the impacts of climate change can differ by regions and the respective populations. In addition to global or multinational studies, detailed investigations at the city or regional level are required to complement our understanding. Such studies are important as they can identify population-specific vulnerabilities and local environmental traits, ultimately supporting tailored public health care measures.

This study examines the impact of extreme ambient temperatures on respiratory-related outpatient treatments and hospital admissions in Augsburg, southern Germany. By analyzing and contrasting these patterns, we aim to offer in-depth insights for the implementation of both immediate action plans during extreme temperature events and long-term adaptation strategies.

## Materials and methods

### Location and data collection

This study conducted a retrospective time series analysis to investigate the effects of extreme ambient temperatures on respiratory health outcomes in Augsburg, Bavaria, southern Germany. For the analysis, we used datasets from four different sources:

1. Hospital admission and outpatient treatment records from the University Clinic of Augsburg (UKA) covering the period from 2006 to 2019. More recent data were not considered due to the onset of the COVID-19 pandemic (SARS-CoV-2) in January 2020. The dataset includes all patients who attended the central emergency department of the UKA and were either hospitalized (*Hospital Admission*) or treated in the outpatient clinic (*Outpatient Treatment*). For each patient, the primary diagnosis is given as a 5-digit ICD-10 (10th revision of the International Classification of Diseases) code. For hospital admissions, both the admission and discharge diagnoses were provided. Additionally, anonymized personal information of the patients, including the postal code, place of residence, age and gender was available. The study was approved by the ethics committee of the Technical University of Munich (TUM) (sign: 2023-112-S-NP) and the ethics committee of the Ludwig Maximilian University of Munich (LMU) (sign: 23-1035).
2. Epidemiological data including the weekly incidence of seasonal influenza for the city and district of Augsburg, sourced from the Robert Koch Institute (RKI). The data are publicly available from the RKI’s database (Robert Koch Institute (RKI), 2025).
3. Meteorological data for Augsburg (Lat.: 48.4253, Lon.: 10.9417) from the German Weather Service (DWD, *Deutscher Wetterdienst*) including the daily mean (T_mean_), minimum (T_min_), and maximum (T_max_) temperatures in [°C] and relative humidity in [%]. The data are publicly available from the Climate Data Center (CDC) (German Weather Service (DWD), 2025).
4. Daily air pollution data in [µg/m^3^] from the Bavarian Environment Agency (Bayerisches Landesamt für Umwelt, LfU) at four different measuring stations in Augsburg (LfU (Latitude (Lat.): 48.3260, Longitude (Lon.): 10.9031), Königsplatz (Lat.: 48.3646, Lon.: 10.8950), Karlstraße (Lat.: 48.3703, Lon.: 10.8963), Bourges-Platz (Lat.: 48.3766, Lon.: 10.8884)) including measurements of PM_10_, NO, NO_2_ and O_3_. The data are publicly available from the measurement archive of the LfU (Bavarian Environment Agency (LfU), 2025).

### Data pre-processing

To ensure data quality and consistency across the involved datasets, various pre-processing steps were applied. For the hospital admissions, only entries with a discharge diagnosis were included in the analysis, as these are considered more reliable than the admission diagnoses. For both outpatient treatments and hospital admissions, cases sharing the same ID were aggregated by concatenating all associated diagnoses. Eventually, we filtered for cases with a primary respiratory diagnosis (ICD-10: J00.xx–J99.xx). Missing values in the daily relative humidity (14 missing val­ues, ~ 0.3%) and air pollution data were imputed via linear interpolation. There were no missing values for the daily temperature data.

### Statistical analysis

For the time series analysis a quasi-Poisson regression with penalized Distributed Lag Non-linear Models (DLNM) (Gasparrini et al. 2017, 2010) was implemented in R (R Core Team 2021) (Version 4.4.1) as a Generalized Additive Model (GAM) using the libraries dlnm (Gasparrini 2011) and mgcv (Wood 2011). DLNMs allow for the estimation of non-linear and delayed effects and are a well-established framework in the field of environmental epidemiology, in particular to model exposure–response relationships between temperature and both mortality (Gasparrini et al. 2015; Iñiguez et al. 2021; Masselot et al. 2023) and morbidity (Martínez-Solanas and Basagaña, 2019; Wen et al. 2024). The model used in this study can be stated as:

$$ \begin{aligned} \log E\left[ {Y_{t} } \right] & = intercept + cb_{Temp} + cb_{Humidity} + cb_{Influenza} \\ & \quad + dow + phday + seas\left( {t,ns\left( {df = n\;per\;year} \right)} \right) \\ \end{aligned} $$

The model includes an intercept representing the baseline level of the outcome ($intercept$) and cross-basis objects to describe the effect of temperature ($cb_{Temp}$), relative humidity ($cb_{Humidity}$) and influenza incidence ($cb_{Influenza}$). Control variables included are the day of the week ($dow$) and public holidays ($phday$) including Christmas (December 24th) and New Year’s Eve (December 31st). We further control for seasonality and long-term trends via a natural spline function over time $(t)$ and $n$ degrees of freedom per year ($seas\left( {t, ns \left( {df = n\; per\; year} \right)} \right)$). A quasi-Poisson distribution was assumed to account for potential over-dispersion (Gasparrini et al. 2010). The GAM was fitted using restricted maximum likelihood (REML) method (Wood 2011).

#### Seasonality modelling

Selecting the appropriate number of degrees of freedom for modelling seasonality is an often-debated issue, as it can strongly influence the effect estimation. Typically, values in the range between six and ten degrees of freedom per year are considered an appropriate trade-off between capturing seasonal trends and leaving enough information to estimate exposure effects (Bhaskaran et al. 2013; Gasparrini et al. 2010; Masselot et al. 2023; Wen et al. 2024). To determine the optimal level of complexity for the seasonality component ($seas\left( {t, ns \left( {df = n\; per\; year} \right)} \right))$ we additionally considered the adjusted version of the *Akaike Information Criterion* (*qAIC)* (Gasparrini et al. 2010). Within the range of four to twelve degrees of freedom per year, the model with the lowest *qAIC* value was selected as the best fit.

#### Cross-basis parametrizations

The variable dimension of the cross-basis objects for temperature and relative humidity was parametrized via a natural spline with fixed knots at the 10th, 50th and 90th percentile of the variable’s distribution. For the influenza incidence, we assumed a linear relationship following Lytras et al. (2019). For all involved cross-basis objects we chose a maximum lag of 21 days, consistent with previous studies (Gasparrini et al. 2015; Martínez-Solanas and Basagaña, 2019; Wen et al. 2024), and a penalized spline with five degrees of freedom for the lag dimension. Similar to previous work by Obermeier et al. (2015) and Gasparrini et al. (2017), we applied a ridge penalty on the cross-basis coefficients imposing a decay towards the maximum lag value, i.e. we specified a diagonal penalty matrix with entries p = [0, 0, 0, 1, 1]^T^. We chose the penalized spline parametrization over the more commonly used fixed knots (e.g. three equally spaced knots in log scale), due to the greater flexibility regarding the knot placement and the tendency of penalized splines to yield smoother and hence more plausible lag structures, with a decay imposed towards the maximum lag.

#### Relative risk, attributable fractions and attributable numbers

The impact of extreme temperature, relative humidity and influenza incidence on respiratory health outcomes are reported as relative risks (RR). From the fitted cross-basis objects we can obtain effect estimates *β*_*x,l*_ for a given exposure *x* and lag *l*, relative to a reference exposure *x*_*0*_ (Gasparrini and Leone 2014). For temperature and relative humidity, we used the median value of their respective distribution within the study period as the reference exposure *x*_*0*_. We chose the median rather than the *Minimum Morbidity Percentile* or the analogous *Minimum Risk Temperature*, which were frequently used in multiple recent studies (Iñiguez et al. 2021; Wen et al. 2024), but seem challenging to interpret when greatly varying between and even within studies (Iñiguez et al. 2021). For the relative risk associated with the influenza incidence, we used zero as the reference *x*_*0*_. The effect estimates *β*_*x,l*_ translate to the relative risk for a given exposure $x$ and lag $l$ via ${\text{RR}}_{x,l} = \exp \left( {\beta_{x, l} } \right)$. We evaluated the relative risk at four different temperature percentiles: *extreme cold* (1st percentile), *moderate cold* (10th percentile), *moderate heat* (90th percentile) and *extreme heat* (99th percentile).

Following the *backward perspective* described by Gasparrini and Leone (2014), the attributable fractions (AF) and attributable numbers (AN) at a given time $t$ and an exposure $x$ can be defined as:

$$ \begin{array}{*{20}c} {{\text{AF}}_{x,t} = 1 - \exp \left( { - \mathop \sum \limits_{{l = l_{0} }}^{L} \beta_{{x_{t - l} ,l}} } \right) } \\ \end{array} \begin{array}{*{20}c} {{\text{AN}}_{x,t} = {\text{AF}}_{x,t} \;{\text{n}}_{t} } \\ \end{array} $$

with ${\text{n}}_t$ being the number of cases at time $t$ whereas $l_{0}$ and $L$ refer to the minimum and maximum of the considered lag values, respectively.

For the calculation of the AF and AN, we used the R function attrdl of the FluMoDL (Lytras 2019) (Version 0.0.3) library, which was slightly adjusted compared to the initial implementation provided by Gasparrini and Leone (2014) and allows to calculate the AF over user-defined sub-periods. In addition to the estimation of the AF across the entire study period, we used this implementation to calculate the AF on a daily basis and for the two sub-periods 2006–2012 and 2013–2019. The AFs for heat and cold were calculated for temperatures greater or equal to the 90th and less or equal the 10th percentile, respectively. To estimate confidence intervals (CI) for the AF, we used an empirical approach drawing random samples (N = 5,000) from an assumed multivariate normal distribution of the estimated cross-basis coefficients and interpreting the range between the 2.5th and 97.5th percentile of the estimated AF values’ distribution as the 95% confidence interval (Gasparrini and Leone 2014).

#### Sensitivity analysis

An extensive sensitivity analysis was performed to assess the robustness of the results, varying modelling assumptions and controlling for additional potential confounders like air pollution (PM_10_, NO, NO_2_ and O_3_) and a heatwave indicator via linear terms added to the main model. A heatwave was defined as a period of at least three consecutive days with the daily maximum temperature exceeding 30 °C (Tomczyk and Sulikowska 2018). Further, two alternative maximum lag values (14 and 28 days), different choices for the degrees of freedom for the seasonality component and alternative parametrizations of the temperature cross-basis function were included.

#### Short- and long-term effect

While we used a maximum lag of 21 days for the cross-basis objects in all involved models, we calculated the cumulative effects of heat and cold across two selected lag intervals to capture both short- and long-term effects. We defined the *short-term cumulative effect* as the aggregated effect up to three days after the exposure, to avoid masking the more immediate effect by potential *harvesting* or *displacement* effects (Bhaskaran et al. 2013; Qiao et al. 2015; Saha et al. 2014; Schwartz et al. 2004). On the other hand, the *long-term cumulative effect* was calculated over the entire 21 days lag period to capture any delayed effects. This differentiation was also applied when calculating the attributable fractions and numbers, i.e. we calculated *short-term AFs/ANs* as well as a *long-term AFs/ANs,* based on the short- (Lag 0–3 days) and long-term effect (Lag 0–21 days), respectively. By this we aimed to give a more thorough and comprehensive picture of the related health burdens.

#### Vulnerable groups

For a more in-depth understanding of the temperature effect and the identification of vulnerable groups, we stratified the analysis using separated models by: different age groups (0–5 years, 6–15 years, 16–64 years and ≥ 64 years), gender, place of residence (urban and rural areas of Augsburg) and some of the most frequent sub-diagnoses in the dataset (ICD-10: J00–J06 (Acute upper respiratory infections), J12–J18 (Pneumonia), J20–J22 (Other acute lower respiratory infections), J44 (COPD)). The place of residence was classified as urban or rural based on the patient’s postal code, as provided in the medical records. Postal codes were grouped according to administrative boundaries: urban areas included zip codes within the city of Augsburg (*Stadt Augsburg*), while rural areas comprised zip codes from the district of Augsburg (*Landkreis Augsburg*). Patients with postal codes from other areas were excluded from the urban–rural stratification. More detailed diagnoses, e.g. the fourth and the fifth digits of the ICD-10 codes, were not considered due to the limited number of cases.

#### Sub-period analysis

We conducted separate analyses for the two sub-periods 2006–2012 and 2013–2019 to identify trends in the climatic conditions and the associated effects on respiratory health. To ensure comparability between the effect estimates, we fixed the knots for the parameterization of the variable dimension in the cross-basis objects for temperature and relative humidity using the percentiles from the overall study period between 2006 and 2019. We also maintained the definitions of moderate/extreme cold and heat described in Sect. "Relative risk, attributable fractions and attributable numbers" based on the temperature percentiles from the entire study period. An additional sub-period analysis was applied to the estimation of attributable fractions and attributable numbers. For this we evaluated the attributions for the sub-periods 2006–2012 and 2013–2019 separately, using the effect estimates from the entire study period to preserve maximum statistical power.

## Results

The time series of daily emergency cases showed a pronounced seasonal pattern for both, outpatient treatments (Figure S1, (A)) and hospital admissions (Figure S1, (B)). While the daily numbers of outpatient treatments exhibited a long-term rising trend, the hospitalizations showed no clear tendency within the study period. The distributions of the daily case numbers approximately followed a Poisson distribution (Figure S2).

Over the full study period, the dataset contained a total of 77,286 cases with a primary respiratory diagnosis, comprising 34,176 outpatient treatments and 43,110 hospital admissions (Table 1). Whereas the majority of outpatient treatments were on account of very young children (0–5 years, 41.9%) and adults (16–64 years, 41.1%), almost half (48.7%) of the hospital admissions involved patients aged 65 years and older. Acute upper respiratory infections (ICD-10: J00–J06) accounted for nearly two-thirds (63.3%) of the total number of outpatient treatments, while pneumonia (ICD-10: J12–J18), other acute lower respiratory infections (ICD-10: J20–J22), and COPD (ICD-10: J44) were the most frequent diagnoses among hospital admissions (Table 1). The distribution of considered sub-diagnoses varied notably across age groups (Figure S3, Figure S4). While pneumonia and COPD were most frequently observed in patients aged 65 years and over, acute upper respiratory infections and other acute lower respiratory infections were more prevalent among young children aged between 0 and 5 years. Note that, due to some cases in the outpatient treatments being associated with multiple primary diagnoses, summing the cases of sub-diagnoses slightly exceeds the total case number.

**Table 1:** Number of outpatient treatments and hospital admissions with respiratory diagnoses between 2006 and 2019

	Outpatient treatments	Hospital admissions
Overall	34,176	43,110
*Gender*		
Female	16,427 (48.1%)	18,481 (42.9%)
Male	17,744 (51.9%)	24,628 (57.1%)
Other/Unknown	5 (< 0.1%)	1 (< 0.1%)
*Age*		
0–5	14,303 (41.9%)	7,303 (16.9%)
6–15	4,548 (13.3%)	2,172 (5.0%)
16–64	14,038 (41.1%)	12,655 (29.4%)
65 and older	1,287 (3.8%)	20,980 (48.7%)
*Diagnoses*		
J00–J06	21,635 (63.3%)	5,214 (12.1%)
J12–J18	1,164 (3.4%)	15,408 (35.7%)
J20–J22	2,555 (7.5%)	6,042 (14.0%)
J44	257 (0.8%)	5,664 (13.1%)
Other	8,748 (25.6%)	10,782 (25.0%)
*Place of residence*		
Augsburg City	15,057 (44.1%)	20,755 (48.1%)
Augsburg District	10,166 (29.7%)	12,587 (29.2%)
Other	8,953 (26.2%)	9,758 (22.6%)

Percentages in parenthesis indicate the proportion within each respective subgroup

Regarding the long-term climate trends, annual mean air temperatures in Augsburg have risen steadily, from 8.2 °C (1961–1990) to 9.0 °C (1991–2020) (German Weather Service (DWD) 2025). Within the study period between 2006 and 2019 an average daily mean temperature of 9.2 °C was observed. July was the warmest month (18.7 °C), while January was the coldest (0.1 °C). An average of 9.5 hot days (T_max_ ≥ 30 °C) and 22.3 frost days (T_max_ < 0 °C) per year were recorded. For our definitions of *extreme cold* (1st percentile) and *extreme heat* (99th percentile) we obtained daily mean temperatures of − 7.8 °C and 23.7 °C, respectively. The overall average daily relative humidity was 82.0%. The reported influenza incidences varied considerably by year, with the highest seasonal peak values observed in November 2009 (66.1 per 100,000 population) and February/March 2019 (20.4 per 100,000 population). Additional descriptive statistics regarding the daily case numbers and environmental variables can be found in Table S1.

### Respiratory health risks from heat and cold exposure

For both treatment settings, we selected a seasonality component with nine degrees of freedom, minimizing the *qAIC* (Figure S5). Our modelling using DLNMs revealed distinct lag structures for the effects of heat and cold on the daily number of respiratory-related treatments.

**Fig. 1:**
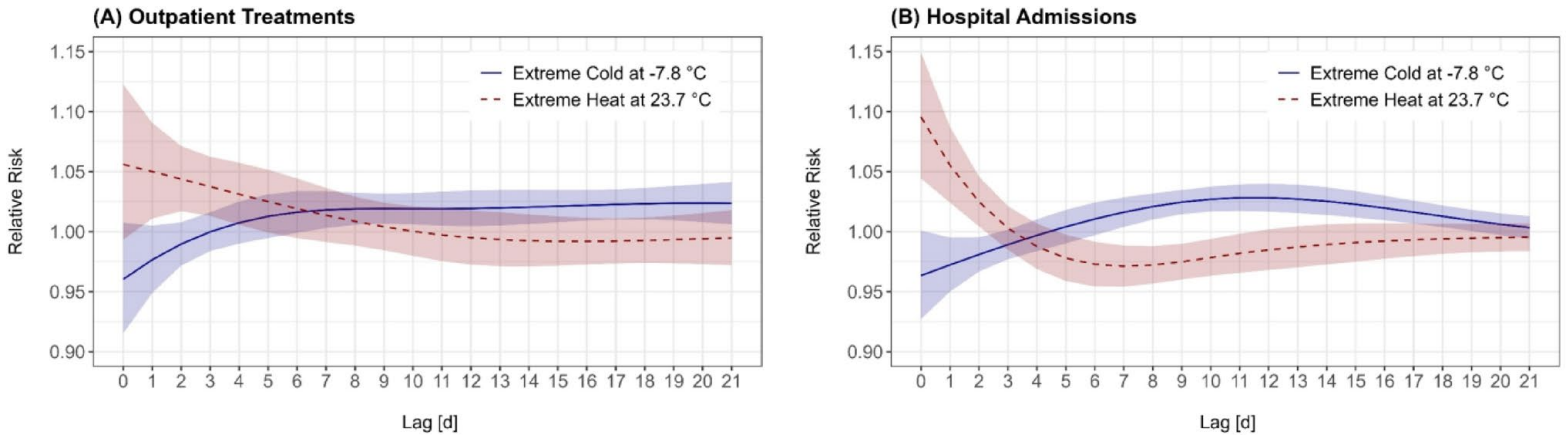
Lag structure of the extreme cold (blue, solid) and extreme heat (red, dashed) effect on outpatient treatments (**A**) and hospital admissions (**B**) due to respiratory conditions. Relative risks (RRs) on the y-axis are shown against the lag days (0–21) on the x-axis. The shaded regions represent the 95% confidence intervals

In case of outpatient treatments, extreme heat led to an immediately increased risk which persisted for up to six days after the exposure, followed by a weak protective effect (Fig. 1A). Conversely, extreme cold resulted in a short-term protective effect that turned into an increased risk after three days, continuing as a sustained long-term elevated risk (Fig. 1A). For hospital admissions, extreme heat exhibited an immediate effect, with an increased risk on the same day and up to three days after the exposure, followed by a prolonged period of significantly reduced risk. In contrast, extreme cold produced a short-term protective effect on the same day of exposure that turned into a significantly elevated risk between one and three weeks after the exposure (Fig. 1B).

**Fig. 2:**
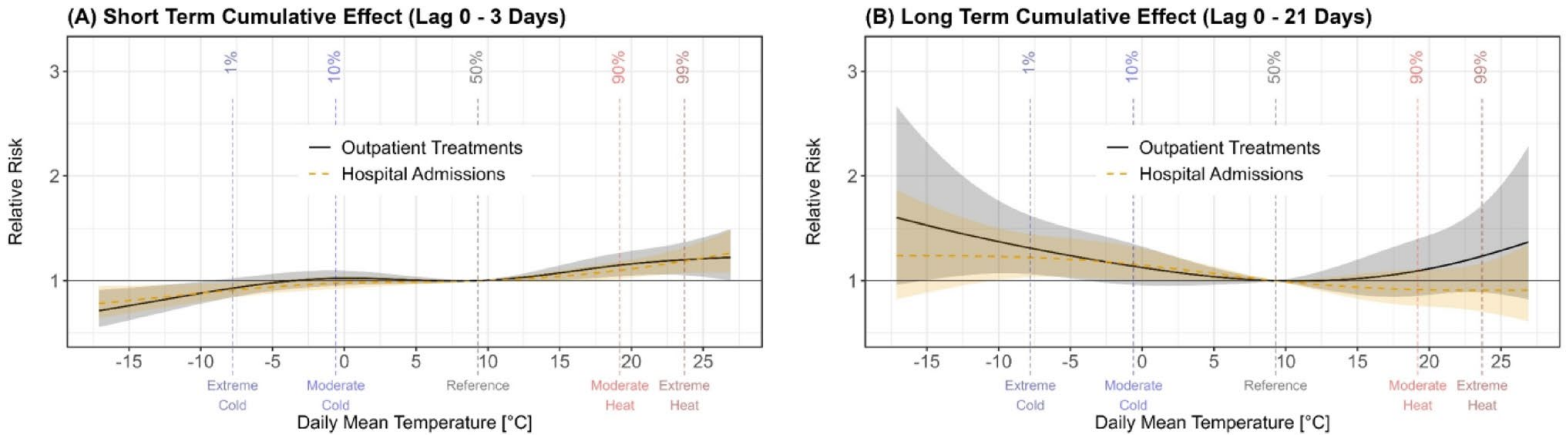
Short- (**A**) and long-term (**B**) cumulative effect reported as relative risk (RR) on the daily number of outpatient treatments (black, solid) and hospital admissions (yellow, dashed) in the observed temperature range. The shaded regions represent the 95% confidence intervals

The *short-term cumulative effect* (Lag: 0–3 days) of extreme heat resulted in a RR of 1.20 (95% CI [1.05, 1.37]) for outpatient treatments and 1.19 (95% CI [1.07, 1.32]) for hospital admissions, while extreme cold led to a RR of 0.93 (95% CI [0.84, 1.02]) for outpatient treatments and 0.91 (95% CI [0.84, 0.98]) for hospital admissions (Fig. 2A). On the other hand, the *long-term cumulative effect* (Lag: 0–21 days) of extreme heat yielded a RR of 1.24 (95% CI [0.89, 1.72]) for outpatient treatments and 0.91 (95% CI [0.70, 1.17]) for hospital admissions, while extreme cold resulted in a RR of 1.31 (95% CI [1.06, 1.62]) for outpatient treatments and 1.22 (95% CI [1.03, 1.45]) for hospital admissions (Fig. 2B).

The stratification by age, gender, place of residence and selected sub-diagnoses revealed notable differences in temperature-related health risks across these groups. For outpatient treatments, the short-term effect of extreme heat was more pronounced in men (RR: 1.26 (95% CI [1.06, 1.49])) compared to women (RR: 1.14 (95% CI [0.95, 1.36])) (Table S2). Very young children (0–5 years) were the most vulnerable to the long-term effect of extreme cold (RR: 1.43 (95% CI [1.05, 1.95])), while people aged between 16 and 64 years had the highest risk increase due to the long-term effect of extreme heat (Table S3). For hospital admissions, women (RR: 1.28 (95% CI [1.10, 1.50]) and individuals aged 65 years and older (RR: 1.26 (95% CI [1.09, 1.46])) were particularly vulnerable to the short-term effect of heat (Table S4). In addition, older adults (≥ 65 years) were the most vulnerable group regarding the long-term effect of extreme cold exposure (RR: 1.36 (95% CI [1.08, 1.72])) (Table S5). For both treatment settings, individuals residing in urban areas showed a higher risk associated with both temperature extremes compared to those living in rural areas.

**Fig. 3:**
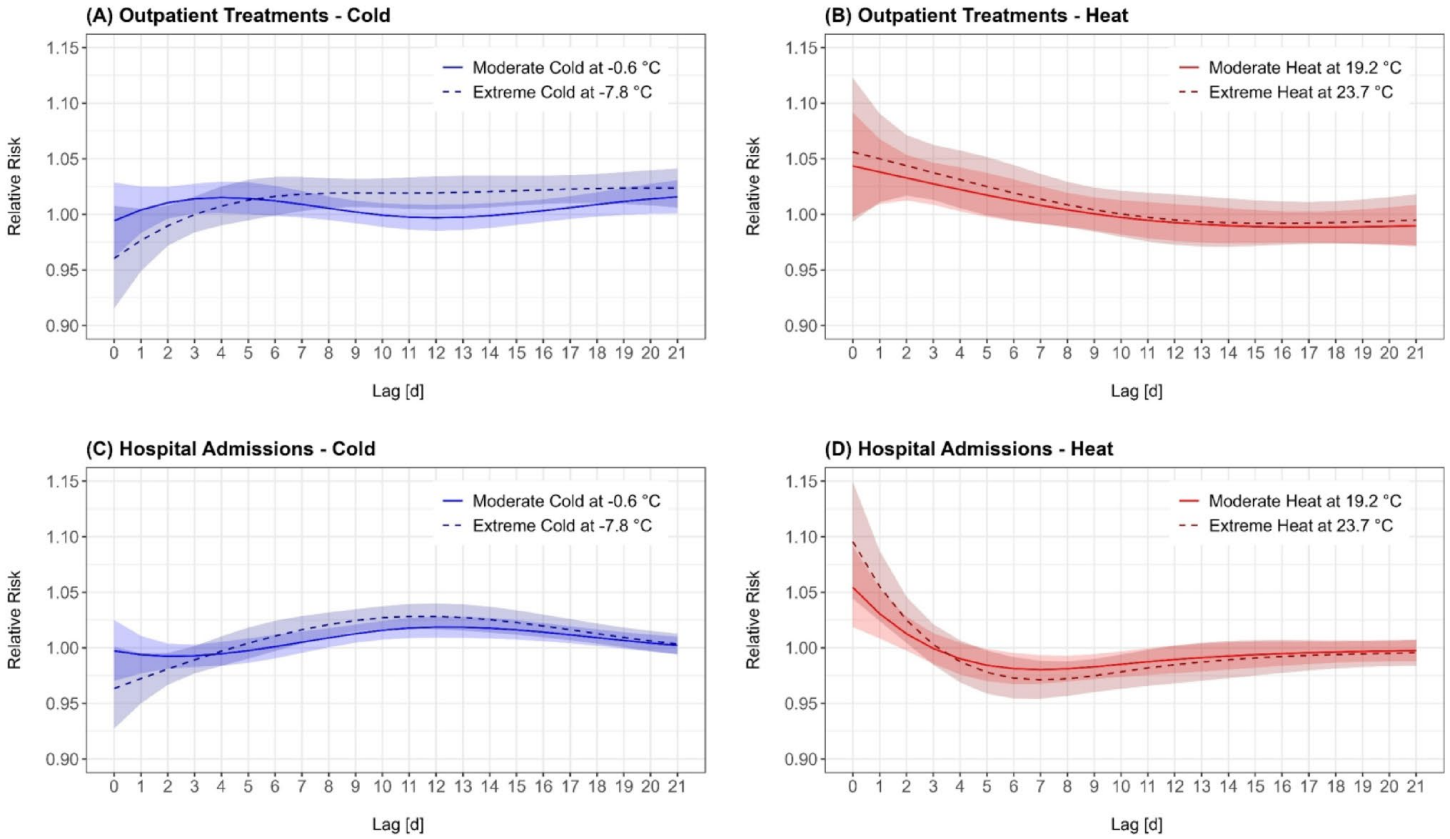
Relative Risk curves showing the effects of moderate (solid lines, light red/blue) and extreme (dashed lines, dark red/blue) cold and heat on respiratory health outcomes. Panels A and B depict the effects of heat and cold on outpatient treatments; panels C and D show the effects on hospital admissions. The shaded regions represent the 95% confidence intervals

We further found differences in the effect patterns of moderate and extreme temperature exposure (Fig. 3). In both treatment settings we observed that the short-term protective effect and the subsequent delayed risk increase were stronger expressed for extreme cold compared to moderate cold (Fig. 3A, C). Comparing the effects of moderate and extreme heat on outpatient treatments, a higher risk across the entire lag period for extreme heat (Fig. 3B) was observed. In case of hospital admissions, extreme heat led to a stronger short-term risk increase, followed by a stronger protective delayed effect compared to moderate heat (Fig. 3D).

To further quantify the public health impact of temperature extremes, we estimated the attributable fractions and attributable numbers of the respiratory-related emergency cases associated with cold and heat, based on both short- and long-term effects. The *short-term AF* (Lag: 0—3 days) due to cold was − 0.15% (95% CI [− 1.25, 0.83]) for outpatient treatments and − 0.72% (95% CI [− 1.61, 0.11]) for hospital admissions (Table 2). In contrast, heat exposure was associated with a *short-term AF* of 1.16% (95% CI [0.41, 1.84]) for outpatient treatments and 0.97% (95% CI [0.38, 1.53]) for hospital admissions. The *long-term AF* (Lag: 0—21 days) due to cold was 2.64% (95% CI [0.27, 4.71]) for outpatient treatments and 2.33% (95% CI [0.45, 3.98]) for hospital admissions (Table 2). For heat, we obtained a *long-term AF* of 1.06% (95% CI [− 0.92, 2.64]) for outpatient treatments and − 0.70% (95% CI [− 2.40, 0.81]) for hospital admissions.

**Table 2:** Attributable fractions (AF in [%]) and attributable numbers (AN) of respiratory related outpatient treatments and hospital admissions associated with cold and heat exposure

	Cold		Heat	
	AF (%)	AN	AF (%)	AN
*Outpatient treatments*				
Short-term (Lag 0–3 days)	− 0.15 (− 1.25, 0.83)	− 51 (− 427, 283)	1.16 (0.41, 1.84)	398 (139, 630)
Long-term (Lag 0–21 days)	2.64 (0.27, 4.71)	901 (92, 1609)	1.06 (− 0.92, 2.64)	361 (− 314, 902)
*Hospital admissions*				
Short-term (Lag 0–3 days)	− 0.72 (− 1.61, 0.11)	− 309 (− 693, 46)	0.97 (0.38, 1.53)	420 (162, 659)
Long-term (Lag 0–21 days)	2.33 (0.45, 3.98)	1002 (193, 1715)	− 0.70 (− 2.40, 0.81)	− 302 (− 1034, 348)

The estimated AFs and ANs are presented separately for short-term (Lag 0–3 days) and the long-term (Lag 0–21 days) effects. The parentheses indicate the 95% confidence intervals

The differences between short-term and long-term effect become particularly apparent when looking at the daily resolution of the attributable numbers, illustrated as an example for the year 2017 (Figure S6). For both treatment settings, an accumulation of frost days was followed by a negative number of short-term attributable cases, i.e. a protective effect (Figure S6, (A), (C)), which turned into significantly positive attributions when considering the long-term effect (Figure S6, (B), (D)). Hot days were followed by positive *short-term ANs* for both hospital admissions and outpatient treatments (Figure S6, (A), (C)), while the long-term protective effect of heat on hospital admissions becomes apparent as negative attributions (Figure S6, (D)).

The sensitivity analysis confirmed the robustness of the main results, with relative risks for both outpatient treatments and hospital admissions remaining largely stable across alternative model specifications, lag structures, temperature parametrizations, and adjustments for air pollution. While some variation was observed—particularly in the *long-term cumulative effect* after cold exposure—the overall effect patterns of temperature extremes remained consistent. The results from the sensitivity analysis can be found in the supplementary material (Table S6, Table S7, Table S8 and Table S9).

### Influence of relative humidity and influenza incidence

For relative humidity, the *short-term cumulative effect* of dry air led to a non-significant risk increase for both treatment settings, outpatient treatments and hospital admissions, with no notable *long-term cumulative effect* observed (Figure S7). On the other hand, the weekly influenza incidence showed significant correlations with both outpatient treatments and hospital admissions (Figure S8). The *long-term cumulative effect* of an incidence of 10 per 100,000 population resulted in a RR of 1.25 (95% CI [1.10, 1.43]) for outpatient treatments and 1.16 (95% CI [1.06, 1.28]) for hospital admissions (Table S10). This corresponded to a *long-term AF* of 2.68% (95% CI [1.27, 3.93]) for outpatient treatments and 1.62% (95% CI [0.60, 2.54]) for hospital admissions (Table S11).

### Effect of the recent rise in temperatures on respiratory health

The temperature distribution over the 14-year study period exhibited a noticeable shift when comparing the two sub-periods, 2006 to 2012 and 2013 to 2019 (Figure S9). In particular, the number of hot days (T_max_ >  = 30 °C) increased, while the number of cold days (T_max_ < 0 °C) decreased (Figure S10). Between 2006 and 2012, an average of seven hot days per year was observed, compared to an average of twelve hot days per year between 2013 and 2019. This shift in the temperature distribution also affected the effect estimations for the two sub-periods.

**Fig. 4:**
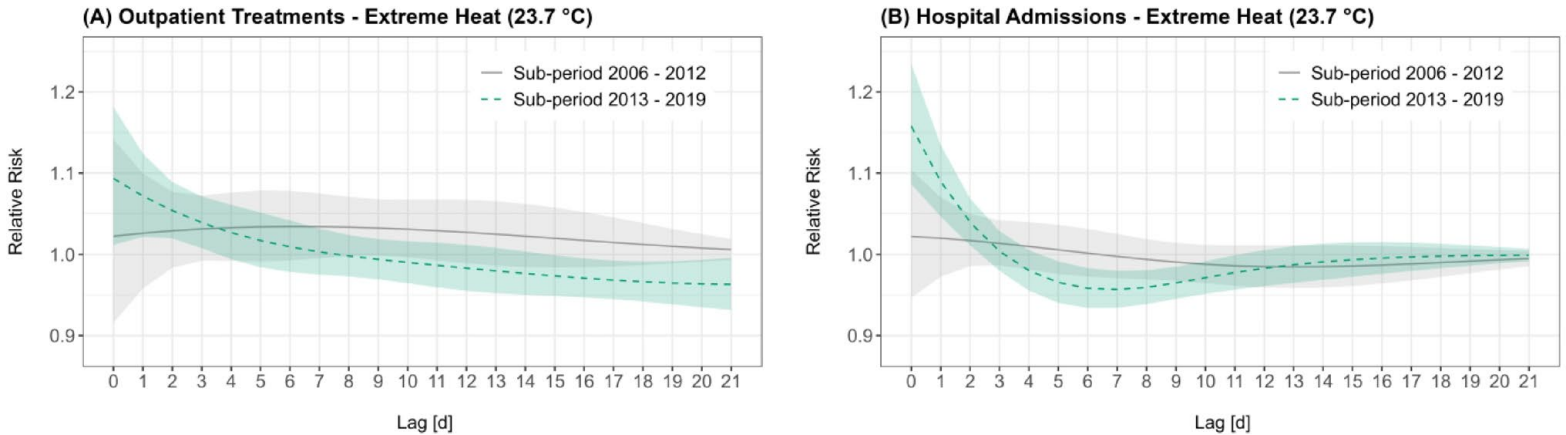
Relative Risk curves showing the effects of extreme heat on outpatient treatments (**A**) and hospital admissions (**B**) for the analysis of the two sub-periods 2006–2012 (light gray, solid) and 2013–2019 (green, dashed). The shaded regions represent the 95% confidence intervals

The same day relative risk associated with extreme heat increased for outpatient treatments (2006–2012: 1.02 (95% CI [0.92, 1.14]), 2013–2019: 1.09 (95% CI [1.01, 1.18])) and hospital admissions (2006–2012: 1.02 (95% CI [0.95, 1.10]), 2013–2019: 1.16 (95% CI [1.09, 1.23])) (Fig. 4). Likewise, the *short-term cumulative effect* (Lag: 0–3 days) increased between the sub-periods, with the relative risk for hospital admissions rising from 1.08 (95% CI [0.91, 1.27]) in 2006–2012 to 1.32 (95% CI [1.15, 1.51]) in 2013–2019. The increase in the short-term relative risk for hospital admissions in the second period was accompanied by a stronger subsequent protective effect, which was only weakly expressed in the first period (Fig. 4B). The *long-term cumulative effect* (Lag: 0–21 days) of extreme heat stayed approximately the same for hospital admissions, while it declined sharply for outpatient treatments (2006–2012, RR: 1.70 (95% CI [0.96, 2.99]), 2013–2019, RR: 0.98 (95% CI [0.65, 1.49])). Details from the sub-period analysis for extreme heat and extreme cold are provided in Table 3.

**Table 3:** Effect estimations for extreme heat and extreme cold for the two subperiods 2006–2012 and 2013–2019 including the same day effect and the cumulative effects over selected lag periods

Extreme cold (T_Mean_ = − 7.8 °C)			
	Lag 0	Lag 0–3	Lag 0–21
*Hospital admissions*			
2006–2012	0.96 (0.91, 1.01)	0.88 (0.79, 0.99)	1.09 (0.86, 1.37)
2013–2019	0.96 (0.91, 1.02)	0.95 (0.84, 1.07)	1.30 (0.97, 1.73)
*Outpatient treatments*			
2006–2012	0.99 (0.92, 1.07)	0.95 (0.82, 1.10)	1.53 (1.11, 2.11)
2013–2019	0.93 (0.87, 0.99)	0.92 (0.80, 1.06)	0.94 (0.68, 1.30)

Extreme heat (T_Mean_ = 23.7 °C)			
	Lag 0	Lag 0–3	Lag 0–21
*Hospital admissions*			
2006–2012	1.02 (0.95, 1.10)	1.08 (0.91, 1.27)	0.93 (0.63, 1.38)
2013–2019	1.16 (1.09, 1.23)	1.32 (1.15, 1.51)	0.95 (0.68, 1.33)
*Outpatient treatments*			
2006–2012	1.02 (0.92, 1.14)	1.11 (0.88, 1.41)	1.70 (0.96, 2.99)
2013–2019	1.09 (1.01, 1.18)	1.28 (1.09, 1.51)	0.98 (0.65, 1.49)

The effects estimates are reported as relative risks for the 1st (extreme cold) and 99th (extreme heat) temperature percentile compared to the median. The parentheses indicate the 95% confidence intervals

The shift in the temperature distribution was also reflected in the number of cases attributed to the two temperature extremes by the model. While the *short-term AN* of cold showed only minor differences between the two sub-periods, the *short-term AN* of heat increased for both outpatient treatments (2006–2012: 127 (95% CI [45, 202]) vs. 2013–2019: 270 (95% CI [95, 428])) and hospital admissions (2006–2012: 166 (95% CI [61, 265]) vs. 2013–2019: 254 (95% CI [101, 394])) (Figure S11 (A), (B)). The *long-term AN* due to cold increased for outpatient treatments (2006–2012: 430 (95% CI [83, 732]) vs. 2013–2019: 472 (95% CI [8, 884])), whereas the attributed hospital admissions decreased (2006–2012: 544 (95% CI [120, 917]) vs. 2013–2019: 459 (95% CI [74, 803])) (Figure S11 (C), (D)). For the *long-term AN* of heat, opposite developments were observed for the two treatment settings. Outpatient treatments showed an increase in attributable cases in the second period (Figure S11 (C)), while hospital admissions became more negative in the second period, indicating a stronger protective *long-term cumulative effect* of extreme heat (Figure S11 (D)). For more details, including a comparison of the AFs for the two sub-periods, see Figure S12, Table S12 and Table S13.

## Discussion

This study provides detailed and novel insights into the impact of extreme ambient temperatures and potential confounders on respiratory health, revealing distinct temporal effect patterns and vulnerabilities for outpatient visits and hospital admissions.

### Heat effect

In short term, heat exacerbates respiratory conditions, particularly asthma and COPD, via inflammatory pathways and increased ozone levels, particulate matter, and direct heat stress (Deng et al. 2020). Previous research has shown that both hospital admissions and outpatient treatments due to respiratory conditions surge during heatwaves (Bujosa Mateu et al. 2024). Our findings confirm this, revealing a pronounced short-term heat effect for both hospital admissions (0 to 3 days after the exposure) and outpatient treatments (0 to 6 days after the exposure). Apart from this common short-term heat effect, we found notable differences in the lag response structures of the two treatment settings. For hospital admissions, the short-term increase of the relative risk is subsequently followed by a significant protective effect, while the latter is only weakly expressed for outpatient treatments. A similar delayed risk reduction after heat exposure for hospitalizations with respiratory diagnoses was recently reported in Sweden by Fonseca-Rodríguez et al. (2021), yielding an overall cumulative protective effect (Lag 0–14 days) for extremely hot and humid temperatures (Fonseca-Rodríguez et al. 2021). This delayed protective effect could be related to *harvesting* or *mortality displacement*, a phenomenon frequently observed in the context of heat-related mortality (Hajat et al. 2005; Qiao et al. 2015; Saha et al. 2014; Schwartz et al. 2004). It suggests that in the case of extreme heat event, an immediate rise in mortality is followed by a subsequent decline, which is explained by the assumption that some of the deaths in vulnerable populations occur earlier than they would have without the heat event (Qiao et al. 2015). These additional deaths could also contribute to the observed reduction of hospital admissions in the days following the heat episode. Further, an analogous *hospitalization displacement* could play a role in the protective effect, as the patients hospitalized during the heat event, could either still be hospitalized in the following days or were presumably discharged in a more stable health condition. This explanation is consistent with the clearly weaker expression of the long-term protective effect of heat on outpatient treatments, where the patients leave the facility after the treatment and are likely examined less thoroughly. Heat prevention programs and early outpatient treatments during the heat season could further enhance this effect. Additionally, deaths and hospitalizations due to other heat-related conditions (e.g. volume depletion or cardiovascular disease) may contribute to the observed *displacement* effect. The comparison of moderate and extreme heat on hospital admissions is also in line with this interpretation, as a stronger immediate effect is followed by a more pronounced reduction in risk (Fig. 3D).

### Cold effect

The influence of extreme cold exposure also exhibited differences in the lag effect structures for the two treatment settings. For outpatient treatments, the increased relative risk peaked earlier (5 to 7 days after the exposure) than for hospital admissions (10 to 12 days after the exposure). This shorter delay could be explained by the higher number of upper respiratory tract infections among outpatient treatments (Figure S3), which typically develop faster compared to diseases of the lower respiratory system, which are more common in hospital admissions (Figure S4). In general, the severity of the symptoms—which may require more time to fully develop—can be expected to be higher for hospitalizations compared to outpatient treatments. The short-term protective effect of extreme cold on hospital admissions followed by a delayed risk increase, peaking at around 10 to 12 days after the exposure is in agreement with the findings from nationwide studies in Spain (Achebak et al. 2024; Iñiguez et al. 2021), which reported similar lagged effects of extreme cold on respiratory-related hospitalizations.

### Vulnerable groups

Identifying vulnerable groups is a crucial step in designing targeted health care interventions, prevention strategies and patient education. Our stratified analysis by age, gender, sub-diagnoses and place of residence provided important insights into the multiple dimensions of potential vulnerabilities. Examining both treatment settings, outpatient treatments and hospital admissions, enabled further differentiation in this context. For outpatient treatments, men appeared particularly vulnerable to extreme heat, while very young children (Age: 0–5 years) were most susceptible to extreme cold. Young children have not fully developed their thermoregulatory ability, making them more sensitive to temperature extremes. This vulnerability aligns with previous research (Xu et al. 2014), although we did not find any evidence on a higher susceptibility to extreme heat in this age group. In hospital admissions, women showed the highest short-term risk in response to extreme heat, while older adults were particularly vulnerable to both extreme cold and heat. This agrees with the findings from Martínez-Solanas and Basagaña (2019), who reported elevated heat-related risks for women and older adults in Spain, and is further supported by previous evidence confirming the vulnerability of older adults to both temperature extremes (Bunker et al. 2016; Masselot et al. 2023). Chronic medical conditions, circulation issues and a weakened immune system are potential physiological explanations for why older adults are highly susceptible to extreme temperatures (Ebi et al. 2021). The common vulnerability to extreme heat for both women and older adults might also be related to the overrepresentation of women in this age group, although the proportion of women among patients aged 65 years and older (44.0%) is only slightly higher than in all hospitalizations (42.9%). In general, the differences in vulnerability patterns between outpatient treatments and hospital admissions are presumably influenced by the demographic characteristics in these two treatment settings (Table 1), but nevertheless underscore the importance of a differentiated assessment of the adverse health outcomes for the effective development and implementation of targeted education and prevention strategies.

### Attributable cases and confounders

Our estimation of the number of outpatient treatments and hospital admissions attributed to cold and heat revealed further insights. The number of cases attributed to cold temperatures clearly exceeds those attributed to heat in both treatment settings. This is in agreement with a recent nation-wide study in Spain, which reported higher attributable fractions for cold (12.6% (95% CI [− 5.7, 27.5])) than for heat (0.2% (95% CI [− 0.3, 0.6])) regarding respiratory related hospital admissions (Iñiguez et al. 2021). This large discrepancy in attributable fractions between heat and cold observed by Iñiguez et al. (2021) is yet also rooted in the reference temperature used for the estimation, located at the 89th temperature percentile. Our results also align well with a recent multi-national study in Europe, which reported that the cold-related excess mortality outweighs the heat-related deaths (Masselot et al. 2023). With regard to our results for the heat effect on hospital admissions, the picture is more complex, as discussed in Sect. "Heat effect". The short-term effect of heat is completely compensated by a long-term protective effect when considering the full 21 days of lag, resulting in an overall negative attributable fraction of − 0.70 (95% CI [− 2.40, 081]). Although the suggested *hospitalization displacement* indicates a possible explanation, the overall protective effect of heat on respiratory health is inconsistent with earlier findings (Iñiguez et al. 2021; Martínez-Solanas and Basagaña, 2019; Wen et al. 2024). In general, the temperate climate in Augsburg might explain why the heat effect is not as pronounced as in southern Europe countries like Spain or Italy.

The influence of relative humidity on respiratory health outcomes is still an unanswered question with ambiguous evidence in the literature (Davis et al. 2016; Fonseca-Rodríguez et al. 2021; Lin et al. 2009). We found an elevated but non-significant relative risk for the *short-term cumulative effect* of very dry air on outpatient treatments and hospital admissions, while there were no notable effects of very humid air. High collinearity between temperature and humidity further complicate a final assessment. As a part of the sensitivity analysis, the daily air pollution concentrations integrated in the regression model as control variables had no notable impact on the estimated temperature effects and hence did not indicate a relevant confounding potential. As known from previous studies, seasonal influenza is an important driver of respiratory health (Fattore et al. 2024; Lytras et al. 2019; Schindler et al. 2024; Wu et al. 2017), yet it is often not controlled for when estimating the effects of temperature on mortality and morbidity. In our study, the attributable fractions for non-zero influenza incidences were similar in magnitude to the *attributions* to cold temperatures, stressing the role of influenza as a relevant confounder in the context of respiratory health. This is also confirmed by a study in Canada that investigated the impact of influenza and other viruses on the weekly number of hospital admissions with a primary respiratory diagnosis, estimating that 4.7% of hospital admissions were attributable to influenza (Schanzer et al. 2018). A study from Greece also found significant effects of influenza incidence and cold temperature on all-cause mortality, yet with a clearly higher number of estimated excess deaths due to cold (74.7 per 100,000 population (95% CI [35.3, 111.7])) compared to influenza (23.6 per 100,000 population (95% CI [17.8, 29.2])) (Lytras et al. 2019). Yet a direct comparison is difficult, since Lytras et al. (2019) considered all-cause mortality rather than respiratory-related. In the context of respiratory health, the impact of influenza is presumably higher.

### Impact of recent warming on respiratory health risks

A comparative analysis of two sub-periods (2006 to 2012 and 2013 to 2019) indicated an impact of the recent rise in temperatures on respiratory health. The number of hot days rose from an average of seven to twelve days per year, correlating with an increase in the short-term effect strength of extreme heat on both treatment settings. The observed change was particularly striking for hospital admissions, with an elevated same day RR of 1.16 (95% CI [1.09, 1.23]) between 2013 and 2019 compared to 1.02 (95% CI [0.95, 1.10]) in 2006 to 2012. The stronger increase in the relative risk for hospital admissions compared to outpatient treatments is presumably related to the different demographic compositions of the two treatment settings. People aged 65 and older make up 48.7% of all hospital admissions (compared to 3.8% of outpatient treatments) and were the most vulnerable to the short-term effect of extreme heat in the overall analysis. The shift in the temperature distribution also affected the number of cases attributed to the short*-*term effect of heat on outpatient treatments and hospital admissions (Figure S11, Figure S12). The estimated outpatient treatments attributed to the short-term effect of heat more than doubled between the two sub-periods.

Our findings indicate that even in a temperate climate like Augsburg, a temperature regime is being approached in which the non-linear effects of extreme heat can lead to a disproportionate increase in respiratory-related diseases and the associated health care utilization. This is also confirmed by a recent study from He et al. (2024), who investigated the associations between exposure to night-time heat and stroke risk in Augsburg from 2006 to 2020 and found similarly alarming trends. Fitting the model with only the data from the more recent sub-period (2013–2020), led to a significant increase in the estimated stroke risk associated with nocturnal heat. The non-linear nature of these health impacts foreshadows the compounding risks associated with climate change, particularly for vulnerable populations such as older adults and individuals with pre-existing conditions. It should nevertheless be kept in mind that our results suggested an overall long-term protective effect of extreme heat on respiratory hospital admissions. This protective effect for hospital admissions is consequently reflected in the estimations of the *long-term cumulative effect* and the associated attributable numbers. Although at first glance these results stand in opposition to an escalating risk for respiratory morbidity accompanying global warming, the delayed protective effect could partly be explained by the suggested *hospitalization displacement* effect discussed in Sect. "Heat effect".

### Strengths and limitations

The study has several limitations, starting with the relatively small population size, resulting in a limited statistical power. The small number of cases further restricts the ability to perform more detailed analyses across different age groups, specific and secondary diagnoses or different sub-periods. For outpatient treatments, the available admission diagnoses are less reliable than discharge diagnoses for hospital admissions. This was also reflected in a small number of cases, with multiple main diagnoses. Further the outpatient treatments exhibited greater variation in the daily cases numbers, mainly related to the day of the week, which complicates the estimation of accurate exposure–response relationships. Changes in the health care policy, such as the provision of additional health care services, cannot be ruled out and could potentially impair the consistency of the time series across the study period, in particular regarding outpatient treatments. Furthermore, the monocentric nature of our study does not ensure transferability to other geographical regions with different climatic or socio-demographic conditions. Nevertheless, our study has several strengths and implications. Firstly, a consistent time series covering 14 years provides a robust dataset for longitudinal analysis. The diagnoses, in particular the discharge diagnoses of the hospital admissions, are detailed and reliable, enhancing the study's credibility. Focusing on cases with a respiratory main diagnosis allows us to capture the cause-specific effect patterns extreme temperatures exert on human health. Datasets from two distinct treatment settings over the same time period allow for further differentiation. Additionally, the small-scale nature of the analysis enables the use of local meteorological and influenza data, which are crucial for accurate assessments.

### Conclusion

Our 14-year time series analysis in Augsburg revealed distinct temporal effect patterns of extreme ambient temperatures on respiratory morbidity, with both outpatient treatments and hospital admissions exhibiting a pronounced short-term heat effect and a delayed long-term cold effect. Interestingly, an overall long-term protective effect of heat was observed for hospital admissions. In the context of this unexpected result, we discussed potential explanations, including a possible *hospitalization displacement* effect. The observed differences in vulnerability and effect patterns for the two treatment settings emphasize the need for differentiated and targeted healthcare strategies. Regarding potential confounders, we found significant relationships between influenza activity and respiratory morbidity, with attributable fractions of similar magnitude to those found for cold temperatures. Eventually, a recent rise in ambient temperature in Augsburg could be linked to a strong increase in outpatient treatments and hospital admissions attributed to the short-term effect of heat, indicating an accelerating health risk associated with global warming. These findings emphasize the urgent need for the implementation of heat action plans and long-term adaptation strategies. The quantification of the impacts of extreme temperatures is crucial for shaping public health programs, urban planning and emergency response strategies designed to lessen the health toll of extreme heat or cold, particularly among vulnerable groups such as older adults and individuals with pre-existing respiratory conditions. While grounded in a local context, the results of our study will not only facilitate the assessment of local health needs and the specific requirements of the Augsburg population, but also contribute detailed insights to an emerging global evidence base on climate-related health risks.

## Supplementary Information

Below is the link to the electronic supplementary material.

Supplementary Material 1

## Data Availability

The authors do not have permission to share data.
