# Supplementary material for: Extreme temperatures, recent warming and seasonal influenza-linking human exposures to respiratory health in southern Germany
Source: Int Arch Occup Environ Health. 2025 Nov 22;98(9-10):997–1012. doi: 10.1007/s00420-025-02179-y (PMC12672813; doi:10.1007/s00420-025-02179-y)
Supplement: Supplementary file 1 — Supplementary Material 1 [file 420_2025_2179_MOESM1_ESM.docx]

**Supplementary Material**

*Extreme temperatures, recent warming and seasonal influenza - linking human exposures to respiratory health in southern Germany*

**International Archives of Occupational and Environmental Health**

Matteo Boser, Daria Luschkova, Monika Seemann, EXTREME Study Group, Claudia Traidl-Hoffmann, and Maria P. Plaza

Corresponding author: Matteo Boser, [matteo.boser@med.uni-augsburg.de](mailto:matteo.boser@med.uni-augsburg.de) Environmental Medicine - Institute of Environmental Medicine and Integrative Health, Faculty of Medicine, University of Augsburg and University Hospital of Augsburg, Stenglinstrasse 2, 86156 Augsburg, Germany

EXTREME study group: Co-author names, affiliations and e-mails in alphabetical order.

| **Name** | **Affiliation** | **E-Mail** |
| --- | --- | --- |
| Monika Seemann | Environmental Medicine - Institute of Environmental Medicine and Integrative Health, Faculty of Medicine, University of Augsburg and University Hospital of Augsburg, Augsburg, Germany | monika.seemann@med.uni-augsburg.de |
| Gertrud Hammel | Faculty of Humanities and Social Sciences, University of Augsburg, Augsburg, Germany | g.hammel@uni-a.de |
| Anna Lang | Environmental Medicine - Institute of Environmental Medicine and Integrative Health, Faculty of Medicine, University of Augsburg and University Hospital of Augsburg, Augsburg, Germany | anna.lang@med.uni-augsburg.de |
| Dr. Julia Sander | Institute for Digital Medicine, University of Augsburg and University Hospital of Augsburg, Augsburg, Germany | julia.sander@nct-suedwest.de |
| Dr. Iñaki Soto Rey | Institute for Digital Medicine, University of Augsburg and University Hospital of Augsburg, Augsburg, Germany | inaki.sotorey@uk-augsburg.de |
| Dr. Markus Wehler | Department of Emergency Medicine and Medicine IV, University Hospital of Augsburg, Augsburg, Germany | markus.wehler@uk-augsburg.de |
| Dr. Katharina Zeiser | Department of General Medicine, University of Augsburg and University Hospital of Augsburg, Augsburg, Germany | katharina.zeiser@med.uni-augsburg.de |

**Table S1** Descriptive statistics on the daily number of respiratory related cases and environmental variables in Augsburg during the study period between 2006 and 2019.

|  | **Mean (SD)** | **Median** | **Min.** | **Max.** | **IQR** |
| --- | --- | --- | --- | --- | --- |
| **Case Numbers** |  |  |  |  |  |
| Outpatient Treatments | 6.7 (5.4) | 5 | 0 | 47 | 6 |
| Hospital Admissions | 8.4 (3.9) | 8 | 0 | 30 | 5 |
| **Environmental Variables** |  |  |  |  |  |
| Mean Temperature [°C] | 9.2 (7.6) | 9.3 | -17.1 | 26.9 | 12.0 |
| Min. Temperature [°C] | 4.0 (6.7) | 4.1 | -22.1 | 18.3 | 10.0 |
| Max. Temperature [°C] | 14.1 (9.1) | 14.4 | -11.2 | 36.9 | 14.5 |
| Relative Humidity [%] | 80.7 (10.7) | 82 | 37 | 100 | 16 |
| PM_10_ [µg / m^3^] | 23.3 (13.9) | 20.6 | 1.7 | 174.0 | 14.0 |
| NO [µg / m^3^] | 27.1 (22.9) | 20.7 | 1.8 | 253.8 | 24.7 |
| NO_2_ [µg / m^3^] | 35.4 (12.3) | 34.6 | 6.0 | 104.9 | 15.9 |
| O_3_ [µg / m^3^] | 44.7 (23.0) | 46.3 | 0.7 | 127.5 | 35.5 |

**Table S2** Short-term (Lag 0 – 3 days) cumulative (extreme) temperature effect on outpatient treatments reported as relative risk (RR) with 95% confidence intervals. Separate RR values for gender, age groups, specific diagnoses and place of residence are reported.

| **Outpatient Treatments** |  |  |  |  |
| --- | --- | --- | --- | --- |
|  | **Extreme Cold**  (T_Mean_ = -7.8) | **Moderate Cold**  (T_Mean_ = 0.6) | **Moderate Heat**  (T_Mean_ = 19.2) | **Extreme Heat**  (T_Mean_ = 23.7) |
| **Overall** | 0.93 (0.84, 1.02) | 1.02 (0.95, 1.10) | 1.15 (1.04, 1.27) | 1.20 (1.05, 1.37) |
|  |  |  |  |  |
| **Gender** |  |  |  |  |
| Female | 0.91 (0.80, 1.04) | 1.06 (0.97, 1.17) | 1.20 (1.05, 1.37) | 1.14 (0.95, 1.36) |
| Male | 0.95 (0.83, 1.08) | 0.99 (0.90, 1.08) | 1.11 (0.97, 1.26) | 1.26 (1.06, 1.49) |
| **Age** |  |  |  |  |
| 0 – 5 | 1.00 (0.87, 1.15) | 1.05 (0.95, 1.17) | 1.05 (0.90, 1.23) | 1.16 (0.94, 1.42) |
| 6 – 15 | - | - | - | - |
| 16 – 64 | 0.87 (0.75, 1.01) | 0.98 (0.88, 1.09) | 1.22 (1.07, 1.40) | 1.19 (0.99, 1.43) |
| 65 and older | - | - | - | - |
| **Diagnoses** |  |  |  |  |
| J00 – J06 | 0.93 (0.82, 1.04) | 1.04 (0.95, 1.13) | 1.12 (0.99, 1.26) | 1.14 (0.97, 1.34) |
| **Place of residence** |  |  |  |  |
| Augsburg City | 0.92 (0.80, 1.05) | 1.06 (0.96, 1.18) | 1.25 (1.09, 1.43) | 1.35 (1.13, 1.62) |
| Augsburg District | 0.84 (0.71, 0.99) | 0.96 (0.85, 1.08) | 1.02 (0.87, 1.20) | 1.05 (0.84, 1.31) |

**Table S3** Long-term (Lag 0 – 21 days) cumulative (extreme) temperature effect on outpatient treatments reported as relative risk (RR) with 95% confidence intervals. Separate RR values for gender, age groups, specific diagnoses and place of residence are reported.

| **Outpatient Treatments** |  |  |  |  |
| --- | --- | --- | --- | --- |
|  | **Extreme Cold**  (T_Mean_ = -7.8) | **Moderate Cold**  (T_Mean_ = 0.6) | **Moderate Heat**  (T_Mean_ = 19.2) | **Extreme Heat**  (T_Mean_ = 23.7) |
| **Overall** | 1.31 (1.06 1.62) | 1.14 (0.96, 1.35) | 1.09 (0.85, 1.40) | 1.24 (0.89, 1.72) |
|  |  |  |  |  |
| **Gender** |  |  |  |  |
| Female | 1.28 (0.97, 1.69) | 1.20 (0.96, 1.50) | 1.22 (0.88, 1.70) | 1.09 (0.70, 1.70) |
| Male | 1.29 (0.98, 1.70) | 1.04 (0.83, 1.29) | 1.01 (0.74, 1.39) | 1.40 (0.91, 2.15) |
| **Age** |  |  |  |  |
| 0 – 5 | 1.43 (1.05, 1.95) | 1.20 (0.94, 1.55) | 0.83 (0.57, 1.20) | 0.82 (0.49, 1.40) |
| 6 – 15 | - | - | - | - |
| 16 – 64 | 1.12 (0.83, 1.52) | 1.02 (0.80, 1.30) | 1.45 (1.03, 2.03) | 1.67 (1.07, 2.62) |
| 65 and older | - | - | - | - |
| **Diagnoses** |  |  |  |  |
| J00 – J06 | 1.40 (1.08, 1.81) | 1.25 (1.01, 1.54) | 1.07 (0.79, 1.45) | 1.12 (0.74, 1.69) |
| **Place of residence** |  |  |  |  |
| Augsburg City | 1.42 (1.06, 1.91) | 1.25 (0.99, 1.59) | 1.25 (0.89, 1.75) | 1.52 (0.96, 2.40) |
| Augsburg District | 0.94 (0.66, 1.32) | 0.88 (0.67, 1.16) | 1.13 (0.76, 1.69) | 1.11 (0.64, 1.93) |

**Table S4** Short-term (Lag 0 – 3 days) cumulative (extreme) temperature effect on hospital admissions reported as relative risk (RR) with 95% confidence intervals. Separate RR values for gender, age groups, specific diagnoses and place of residence are reported.

| **Hospital Admissions** |  |  |  |  |
| --- | --- | --- | --- | --- |
|  | **Extreme Cold**  (T_Mean_ = -7.8) | **Moderate Cold**  (T_Mean_ = 0.6) | **Moderate Heat**  (T_Mean_ = 19.2) | **Extreme Heat**  (T_Mean_ = 23.7) |
| **Overall** | 0.91 (0.84, 0.98) | 0.98 (0.92, 1.03) | 1.10 (1.02, 1.19) | 1.19 (1.07, 1.32) |
|  |  |  |  |  |
| **Gender** |  |  |  |  |
| Female | 0.91 (0.81, 1.03) | 0.97 (0.89, 1.06) | 1.17 (1.04, 1.31) | 1.28 (1.10, 1.50) |
| Male | 0.91 (0.82, 1.01) | 0.98 (0.91, 1.06) | 1.05 (0.96, 1.16) | 1.13 (0.99, 1.29) |
| **Age** |  |  |  |  |
| 0 – 5 | 0.87 (0.73, 1.05) | 0.98 (0.86, 1.13) | 1.03 (0.85, 1.26) | 1.12 (0.83, 1.50) |
| 6 – 15 | - | - | - | - |
| 16 – 64 | 0.91 (0.79, 1.06) | 0.96 (0.86, 1.06) | 0.98 (0.86, 1.12) | 1.07 (0.90, 1.28) |
| 65 and older | 0.95 (0.85, 1.07) | 1.00 (0.92, 1.08) | 1.19 (1.07, 1.32) | 1.26 (1.09, 1.46) |
| **Diagnoses** |  |  |  |  |
| J12 – J18 | 0.91 (0.80, 1.04) | 0.99 (0.90, 1.08) | 1.12 (0.99, 1.27) | 1.21 (1.02, 1.43) |
| J20 – J22 | 0.99 (0.82, 1.19) | 0.97 (0.84, 1.12) | 1.02 (0.82, 1.26) | 1.35 (0.99, 1.83) |
| J44 | 0.80 (0.64, 1.00) | 0.99 (0.84, 1.15) | 1.27 (1.03, 1.56) | 1.34 (1.02, 1.75) |
| **Place of residence** |  |  |  |  |
| Augsburg City | 0.97 (0.87, 1.09) | 0.99 (0.91, 1.08) | 1.12 (1.00, 1.24) | 1.23 (1.06, 1.42) |
| Augsburg District | 0.86 (0.75, 1.00) | 0.96 (0.86, 1.06) | 1.11 (0.97, 1.27) | 1.13 (0.94, 1.37) |

**Table S5** Long-term (Lag 0 – 21 days) cumulative (extreme) temperature effect on hospital admissions reported as relative risk (RR) with 95% confidence intervals. Separate RR values for gender, age groups, specific diagnoses and place of residence are reported.

| **Hospital Admissions** |  |  |  |  |
| --- | --- | --- | --- | --- |
|  | **Extreme Cold**  (T_Mean_ = -7.8) | **Moderate Cold**  (T_Mean_ = 0.6) | **Moderate Heat**  (T_Mean_ = 19.2) | **Extreme Heat**  (T_Mean_ = 23.7) |
| **Overall** | 1.22 (1.03, 1.45) | 1.16 (1.01, 1.33) | 0.91 (0.76, 1.10) | 0.91 (0.70, 1.17) |
|  |  |  |  |  |
| **Gender** |  |  |  |  |
| Female | 1.23 (0.96, 1.57) | 1.10 (0.91, 1.34) | 0.97 (0.73, 1.28) | 1.06 (0.72, 1.55) |
| Male | 1.21 (0.97, 1.52) | 1.18 (0.99, 1.41) | 0.87 (0.68, 1.12) | 0.82 (0.59, 1.15) |
| **Age** |  |  |  |  |
| 0 – 5 | 1.43 (0.98, 2.09) | 1.26 (0.92, 1.72) | 1.40 (0.86, 2.28) | 1.04 (0.50, 2.17) |
| 6 – 15 | - | - | - | - |
| 16 – 64 | 1.04 (0.75, 1.43) | 1.09 (0.85, 1.40) | 0.65 (0.46, 0.90) | 0.66 (0.42, 1.01) |
| 65 and older | 1.36 (1.08, 1.72) | 1.19 (0.99, 1.44) | 0.93 (0.72, 1.21) | 0.89 (0.63, 1.27) |
| **Diagnoses** |  |  |  |  |
| J12 – J18 | 1.34 (1.02, 1.75) | 1.14 (0.92, 1.42) | 0.89 (0.66, 1.21) | 0.85 (0.56, 1.29) |
| J20 – J22 | 1.37 (0.91, 2.07) | 1.23 (0.87, 1.73) | 0.93 (0.55, 1.58) | 0.85 (0.39, 1.86) |
| J44 | 1.26 (0.79, 1.99) | 1.38 (0.96, 1.96) | 0.87 (0.53, 1.43) | 1.10 (0.57, 2.10) |
| **Place of residence** |  |  |  |  |
| Augsburg City | 1.43 (1.13, 1.81) | 1.21 (1.00, 1.46) | 0.95 (0.73, 1.24) | 0.98 (0.68, 1.41) |
| Augsburg District | 1.03 (0.76, 1.40) | 1.17 (0.91, 1.50) | 0.75 (0.54, 1.06) | 0.63 (0.40, 1.01) |

**Table S6** Results from the sensitivity analysis in the short-term cumulative effect (Lag 0 – 3 days) of (extreme) ambient temperatures on outpatient treatments reported as relative risk (RR). The parentheses contain the 95% confidence intervals.

| **Outpatient Treatments** |  |  |  |  |
| --- | --- | --- | --- | --- |
|  | **Extreme Cold**  (T_Mean_ = -7.8) | **Moderate Cold**  (T_Mean_ = 0.6) | **Moderate Heat**  (T_Mean_ = 19.2) | **Extreme Heat**  (T_Mean_ = 23.7) |
| **Main model** | 0.93 (0.84, 1.02) | 1.02 (0.95, 1.10) | 1.15 (1.04, 1.27) | 1.20 (1.05, 1.37) |
| **Lag dimension of cross-basis** |  |  |  |  |
| Max. Lag: 14 d | 0.87 (0.80, 0.96) | 1.00 (0.94, 1.08) | 1.18 (1.07, 1.30) | 1.21 (1.07, 1.38) |
| Max. Lag: 28 d | 0.96 (0.88, 1.06) | 1.05 (0.98, 1.13) | 1.14 (1.03, 1.25) | 1.18 (1.03, 1.34) |
| 3 knots equally in log space | 0.90 (0.82, 1.00) | 1.01 (0.94, 1.09) | 1.15 (1.04. 1.27) | 1.22 (1.06, 1.39) |
| **Variable dimension of cross-basis** |  |  |  |  |
| Penalized Spline with 5 degrees of freedom | 0.93 (0.85, 1.01) | 0.99 (0.94, 1.05) | 1.11 (1.03, 1.19) | 1.21 (1.08, 1.35) |
| Natural Spline with knots at (10, 75, 90) | 0.92 (0.84, 1.02) | 1.00 (0.93, 1.07) | 1.10 (0.99, 1.21) | 1.19 (1.04, 1.36) |
| Double Threshold with thresholds at the median | 0.93 (0.84, 1.03) | 0.96 (0.91, 1.02) | 1.15 (1.05, 1.26) | 1.22 (1.07, 1.40) |
| **Seasonality component** |  |  |  |  |
| 7 degrees of freedom / year | 0.85 (0.77, 0.93) | 0.94 (0.88, 1.01) | 1.07 (0.98, 1.17) | 1.15 (1.02, 1.31) |
| 8 degrees of freedom / year | 0.88 (0.80, 0.96) | 0.98 (0.91, 1.05) | 1.10 (1.00, 1.21) | 1.20 (1.05, 1.37) |
| 10 degrees of freedom / year | 0.96 (0.86, 1.06) | 1.04 (0.97, 1.12) | 1.13 (1.03, 1.25) | 1.17 (1.03, 1.34) |
| 11 degrees of freedom / year | 0.97 (0.87, 1.07) | 1.05 (0.97, 1.13) | 1.15 (1.04, 1.27) | 1.23 (1.07, 1.41) |
| **Exposure variables** |  |  |  |  |
| Minimum Temperature | 0.98 (0.89, 1.07) | 1.05 (0.99, 1.12) | 1.13 (1.03, 1.24) | 1.19 (1.05. 1.35) |
| Maximum Temperature | 0.88 (0.80, 0.97) | 0.98 (0.91, 1.05) | 1.13 (1.04, 1.24) | 1.20 (1.05, 1.37) |
| Heatwave Control Variable | 0.93 (0.84, 1.02) | 1.02 (0.95, 1.10) | 1.15 (1.04, 1.27) | 1.20 (1.04, 1.38) |
| PM_10_ Control | 0.90 (0.81, 1.00) | 1.01 (0.94, 1.09) | 1.14 (1.03, 1.26) | 1.19 (1.04, 1.35) |
| O_3_ Control | 0.93 (0.84, 1.02) | 1.02 (0.95, 1.10) | 1.15 (1.04, 1.27) | 1.20 (1.05, 1.37) |
| NO Control | 0.95 (0.86, 1.05) | 1.03 (0.96, 1.11) | 1.15 (1.04, 1.26) | 1.20 (1.05, 1.36) |
| NO_2_ Control | 0.95 (0.86, 1.05) | 1.03 (0.96, 1.11) | 1.15 (1.05, 1.27) | 1.21 (1.06, 1.38) |
| No Influenza | 0.93 (0.84, 1.02) | 1.03 (0.95, 1.10) | 1.15 (1.04, 1.26) | 1.20 (1.05, 1.37) |

**Table S7** Results from the sensitivity analysis in the short-term cumulative effect (Lag 0 – 3 days) of (extreme) ambient temperatures on hospital admissions reported as relative risk (RR). The parentheses contain the 95% confidence intervals.

| **Hospital Admissions** |  |  |  |  |
| --- | --- | --- | --- | --- |
|  | **Extreme Cold**  (T_Mean_ = -7.8) | **Moderate Cold**  (T_Mean_ = 0.6) | **Moderate Heat**  (T_Mean_ = 19.2) | **Extreme Heat**  (T_Mean_ = 23.7) |
| **Main model** | 0.91 (0.84, 0.98) | 0.98 (0.92, 1.03) | 1.10 (1.02, 1.19) | 1.19 (1.07, 1.32) |
| **Lag dimension of cross-basis** |  |  |  |  |
| Max. Lag: 14 d | 0.89 (0.82, 0.96) | 0.96 (0.91, 1.02) | 1.11 (1.03, 1.19) | 1.21 (1.09, 1.33) |
| Max. Lag: 28 d | 0.90 (0.83, 0.97) | 0.96 (0.91, 1.02) | 1.11 (1.02, 1.19) | 1.17 (1.06, 1.30) |
| 3 knots equally in log space | 0.91 (0.84, 0.99) | 0.97 (0.92, 1.03) | 1.11 (1.02, 1.19) | 1.19 (1.07, 1.32) |
| **Variable dimension of cross-basis** |  |  |  |  |
| Penalized Spline with 5 degrees of freedom | 0.89 (0.83, 0.95) | 0.93 (0.90, 0.97) | 1.07 (1.03, 1.11) | 1.11 (1.05 ,1.17) |
| Natural Spline with knots at (10, 75, 90) | 0.91 (0.84, 0.98) | 0.97 (0.92, 1.03) | 1.10 (1.02, 1.19) | 1.18 (1.06, 1.31) |
| Double Threshold with thresholds at the median | 0.92 (0.85, 1.00) | 0.95 (0.91, 1.00) | 1.12 (1.04, 1.20) | 1.18 (1.06, 1.30) |
| **Seasonality component** |  |  |  |  |
| 7 degrees of freedom / year | 0.86 (0.80, 0.92) | 0.93 (0.88, 0.98) | 1.08 (1.01, 1.16) | 1.19 (1.07, 1.31) |
| 8 degrees of freedom / year | 0.88 (0.81, 0.95) | 0.95 (0.90, 1.01) | 1.08 (1.01, 1.16) | 1.18 (1.07, 1.31) |
| 10 degrees of freedom / year | 0.93 (0.86, 1.01) | 0.98 (0.92, 1.04) | 1.09 (1.01, 1.17) | 1.17 (1.05, 1.30) |
| 11 degrees of freedom / year | 0.93 (0.86, 1.01) | 0.98 (0.92, 1.04) | 1.13 (1.04, 1.22) | 1.21 (1.09, 1.35) |
| **Exposure variables** |  |  |  |  |
| Minimum Temperature | 0.92 (0.86, 1.00) | 0.97 (0.93, 1.02) | 1.03 (0.96, 1.11) | 1.11 (1.01, 1.23) |
| Maximum Temperature | 0.91 (0.84, 0.98) | 0.96 (0.91, 1.03) | 1.09 (1.02, 1.16) | 1.18 (1.06, 1.31) |
| Heatwave Control Variable | 0.91 (0.84, 0.98) | 0.98 (0.92, 1.03) | 1.10 (1.02, 1.19) | 1.19 (1.07, 1.33) |
| PM_10_ Control | 0.89 (0.82, 0.97) | 0.97 (0.92, 1.03) | 1.09 (1.01, 1.18) | 1.18 (1.06, 1.31) |
| O_3_ Control | 0.90 (0.83, 0.97) | 0.97 (0.92, 1.03) | 1.11 (1.03, 1.19) | 1.21 (1.09, 1.34) |
| NO Control | 0.91 (0.84, 0.99) | 0.98 (0.92, 1.04) | 1.10 (1.02, 1.19) | 1.19 (1.07, 1.32) |
| NO_2_ Control | 0.90 (0.83, 0.98) | 0.97 (0.92, 1.03) | 1.10 (1.02, 1.18) | 1.19 (1.07, 1.32) |
| No Influenza | 0.90 (0.84, 0.98) | 0.97 (0.92, 1.03) | 1.10 (1.02, 1.18) | 1.19 (1.07, 1.32) |

**Table S8** Results from the sensitivity analysis in the long-term cumulative effect (Lag 0 – 21 days) of (extreme) ambient temperatures on outpatient treatments reported as relative risk (RR). The parentheses contain the 95% confidence intervals.

| **Outpatient Treatments** |  |  |  |  |
| --- | --- | --- | --- | --- |
|  | **Extreme Cold**  (T_Mean_ = -7.8) | **Moderate Cold**  (T_Mean_ = 0.6) | **Moderate Heat**  (T_Mean_ = 19.2) | **Extreme Heat**  (T_Mean_ = 23.7) |
| **Main model** | 1.31 (1.06, 1.62) | 1.14 (0.96, 1.35) | 1.09 (0.85, 1.40) | 1.24 (0.89, 1.72) |
| **Lag dimension of cross-basis** |  |  |  |  |
| Max. Lag: 14 d | 0.99 (0.85, 1.16) | 1.03 (0.91, 1.16) | 1.27 (1.06, 1.52) | 1.36 (1.06, 1.73) |
| Max. Lag: 28 d | 1.81 (1.37, 2.38) | 1.42 (1.13, 1.78) | 1.03 (0.75, 1.42) | 1.10 (0.71, 1.72) |
| 3 knots equally in log space | 1.41 (1.13, 1.77) | 1.19 (0.99, 1.42) | 1.07 (0.83, 1.39) | 1.16 (0.82, 1.65) |
| **Variable dimension of cross-basis** |  |  |  |  |
| Penalized Spline with 5 degrees of freedom | 1.31 (1.06, 1.63) | 1.11 (0.95, 1.30) | 1.06 (0.87, 1.31) | 1.20 (0.87, 1.66) |
| Natural Spline with knots at (10, 75, 90) | 1.29 (1.04, 1.59) | 1.08 (0.91, 1.27) | 0.95 (0.73, 1.24) | 1.29 (0.92, 1.81) |
| Double Threshold with thresholds at the median | 1.39 (1.11, 1.74) | 1.21 (1.06, 1.38) | 1.18 (0.93, 1.49) | 1.27 (0.90, 1.78) |
| **Seasonality component** |  |  |  |  |
| 7 degrees of freedom / year | 0.92 (0.78, 1.10) | 0.87 (0.75, 1.01) | 0.85 (0.69, 1.05) | 0.93 (0.69, 1.26) |
| 8 degrees of freedom / year | 1.06 (0.88, 1.28) | 0.97 (0.83, 1.14) | 0.88 (0.71, 1.10) | 1.10 (0.80, 1.51) |
| 10 degrees of freedom / year | 1.42 (1.13, 1.78) | 1.20 (1.00, 1.45) | 1.07 (0.83, 1.37) | 1.06 (0.75, 1.49) |
| 11 degrees of freedom / year | 1.43 (1.13, 1.82) | 1.22 (1.00, 1.49) | 1.12 (0.84, 1.49) | 1.36 (0.93, 1.99) |
| **Exposure variables** |  |  |  |  |
| Minimum Temperature | 1.36 (1.10, 1.69) | 1.03 (0.88, 1.22) | 1.00 (0.78, 1.29) | 1.11 (0.80, 1.53) |
| Maximum Temperature | 1.19 (0.98, 1.46) | 1.12 (0.94, 1.34) | 1.15 (0.92, 1.43) | 1.33 (0.95, 1.87) |
| Heatwave Control Variable | 1.31 (1.06, 1.62) | 1.14 (0.96, 1.35) | 1.09 (0.85, 1.40) | 1.24 (0.89, 1.72) |
| PM_10_ Control | 1.27 (1.02, 1.57) | 1.13 (0.95, 1.34) | 1.09 (0.85, 1.39) | 1.23 (0.88, 1.71) |
| O_3_ Control | 1.31 (1.06, 1.62) | 1.14 (0.96, 1.35) | 1.09 (0.86, 1.40) | 1.24 (0.89, 1.73) |
| NO Control | 1.36 (1.10, 1.68) | 1.16 (0.98, 1.38) | 1.09 (0.85, 1.40) | 1.23 (0.89, 1.72) |
| NO_2_ Control | 1.37 (1.10, 1.70) | 1.16 (0.98, 1.38) | 1.09 (0.86, 1.40) | 1.23 (0.88, 1.72) |
| No Influenza | 1.39 (1.12 ,1.71) | 1.21 (1.02, 1.44) | 1.04 (0.81, 1.34) | 1.20 (0.86, 1.68) |

**Table S9** Results from the sensitivity analysis in the long-term cumulative effect (Lag 0 – 21 days) of (extreme) ambient temperatures on hospital admissions reported as relative risk (RR). The parentheses contain the 95% confidence intervals.

| **Hospital Admissions** |  |  |  |  |
| --- | --- | --- | --- | --- |
|  | **Extreme Cold**  (T_Mean_ = -7.8) | **Moderate Cold**  (T_Mean_ = 0.6) | **Moderate Heat**  (T_Mean_ = 19.2) | **Extreme Heat**  (T_Mean_ = 23.7) |
| **Main model** | 1.22 (1.03, 1.45) | 1.16 (1.01, 1.33) | 0.91 (0.76, 1.10) | 0.91 (0.70, 1.17) |
| **Lag dimension of cross-basis** |  |  |  |  |
| Max. Lag: 14 d | 1.10 (0.97, 1.25) | 1.08 (0.97, 1.19) | 0.97 (0.85, 1.12) | 0.98 (0.81, 1.19) |
| Max. Lag: 28 d | 1.30 (1.04, 1.61) | 1.19 (1.00, 1.42) | 0.97 (0.77, 1.24) | 0.88 (0.63, 1.22) |
| 3 knots equally in log space | 1.19 (0.99, 1.42) | 1.13 (0.98, 1.30) | 0.91 (0.75, 1.11) | 0.90 (0.68, 1.18) |
| **Variable dimension of cross-basis** |  |  |  |  |
| Penalized Spline with 5 degrees of freedom | 1.21 (1.05, 1.40) | 1.12 (1.03, 1.21) | 0.90 (0.82, 0.97) | 0.85 (0.75, 0.96) |
| Natural Spline with knots at (10, 75, 90) | 1.21 (1.02, 1.43) | 1.14 (1.00, 1.29) | 0.88 (0.72, 1.08) | 0.91 (0.70, 1.18) |
| Double Threshold with thresholds at the median | 1.25 (1.05, 1.49) | 1.14 (1.03, 1.26) | 0.93 (0.78, 1.11) | 0.90 (0.70, 1.17) |
| **Seasonality component** |  |  |  |  |
| 7 degrees of freedom / year | 0.98 (0.85, 1.14) | 0.97 (0.86, 1.09) | 0.88 (0.75, 1.04) | 0.87 (0.69, 1.09) |
| 8 degrees of freedom / year | 1.07 (0.92, 1.25) | 1.05 (0.92, 1.19) | 0.85 (0.72, 1.01) | 0.87 (0.68, 1.10) |
| 10 degrees of freedom / year | 1.27 (1.06, 1.52) | 1.16 (1.00, 1.34) | 0.87 (0.72, 1.05) | 0.82 (0.64, 1.07) |
| 11 degrees of freedom / year | 1.26 (1.04, 1.52) | 1.17 (1.00, 1.37) | 1.04 (0.84, 1.29) | 1.00 (0.75, 1.32) |
| **Exposure variables** |  |  |  |  |
| Minimum Temperature | 1.18 (1.00, 1.41) | 1.06 (0.93, 1.21) | 0.79 (0.65, 0.95) | 0.78 (0.61, 1.00) |
| Maximum Temperature | 1.18 (1.01, 1.39) | 1.15 (1.00, 1.32) | 0.95 (0.80, 1.12) | 0.94 (0.72, 1.22) |
| Heatwave Control Variable | 1.22 (1.03, 1.45) | 1.16 (1.01, 1.33) | 0.91 (0.76, 1.10) | 0.91 (0.70, 1.17) |
| PM_10_ Control | 1.20 (1.01, 1.42) | 1.15 (1.00, 1.32) | 0.91 (0.76, 1.10) | 0.90 (0.70, 1.16) |
| O_3_ Control | 1.21 (1.02, 1.43) | 1.15 (1.01, 1.32) | 0.92 (0.76, 1.11) | 0.92 (0.71, 1.18) |
| NO Control | 1.23 (1.04, 1.46) | 1.16 (1.01, 1.33) | 0.91 (0.76, 1.10) | 0.91 (0.70, 1.17) |
| NO_2_ Control | 1.21 (1.02, 1.44) | 1.15 (1.01, 1.32) | 0.91 (0.76, 1.10) | 0.91 (0.70, 1.17) |
| No Influenza | 1.24 (1.05, 1.46) | 1.18 (1.03, 1.34) | 0.91 (0.76, 1.09) | 0.90 (0.70, 1.16) |

**Table S10** Short- and long-term cumulative effect of the influenza incidence on the daily number of outpatient treatments and hospital admissions reported as relative risk. The parentheses contain the 95% confidence intervals.

|  | | **Influenza incidence increase by 10 / 100.000** |
| --- | --- | --- |
| **Short-Term Effect (Lag 0 – 3 days)** |  | |
| **Outpatient Treatments** | 1.17 (1.11, 1.25) | |
| **Hospital Admissions** | 1.07 (1.02, 1.12) | |
| **Long-Term Effect (Lag 0 – 21 days)** | | |
| **Outpatient Treatments** | 1.25 (1.10, 1.43) | |
| **Hospital Admissions** | 1.16 (1.06, 1.28) | |

**Table S11** Attributable fractions and attributable numbers associated with an increased influenza incidence based on the short- and long-term cumulative effect. The parentheses contain the 95% confidence intervals.

|  | **Attributable Fraction [%]** | **Attributable Numbers** |
| --- | --- | --- |
| **Short-Term Effect (Lag 0 - 3 days)** |  |  |
| **Outpatient Treatments** | 1.96 (1.28, 2.60)  0.75 (0.19, 1.29) | 671 (439, 890) |
| **Hospital Admissions** |  | 322 (81, 554) |
| **Long-Term Effect (Lag 0 – 21 days)** | | |
| **Outpatient Treatments** | 2.68 (1.27, 3.93) | 915 (432, 1342) |
| **Hospital Admissions** | 1.62 (0.60, 2.54) | 700 (257, 1093) |

**Table S12** Short-term (Lag 0 – 3 days) attributable fraction (AF) and attributable number (AN) of cold and heat on outpatient treatments and hospital admissions. The estimated AFs and ANs are shown for the entire study period and the two sub-periods 2006 – 2012 and 2013 – 2019. The parentheses contain the 95% confidence intervals.

|  | **Cold** | | | **Heat** | | |
| --- | --- | --- | --- | --- | --- | --- |
| **Outpatient Treatments** | **AF [%]** | **AN** | **AF [%]** | | **AN** |  |
| Overall | -0.15 (-1.25, 0.83) | -51 (-427, 283) | 1.16 (0.41, 1.84) | | 398 (139, 630) |  |
| 2006 - 2012 | -0.31 (-1.55, 0.80) | -39 (-198, 103) | 1.00 (0.35, 1.58) | | 127 (45, 202) |  |
| 2013 - 2019 | -0.06 (-1.07, 0.86) | -12 (-228, 185) | 1.27 (0.44, 2.00) | | 270 (95, 428) |  |
| **Hospital Admissions** | **AF [%]** | **AN** | **AF [%]** | | **AN** |  |
| Overall | -0.72 (-1.61, 0.11) | -309 (-693, 46) | 0.97 (0.38, 1.53) | | 420 (162, 659) |  |
| 2006 - 2012 | -0.85 (-1.84, 0.05) | -174 (-376, 10) | 0.81 (0.30, 1.30) | | 166 (61, 265) |  |
| 2013 - 2019 | -0.59 (-1.41, 0.16) | -134 (-320, 37) | 1.12 (0.45, 1.74) | | 254 (101, 394) |  |

**Table S13** Long-term (Lag 0 – 21 days) attributable fraction (AF) and attributable number (AN) of cold and heat on outpatient treatments and hospital admissions. The estimated AFs and ANs are shown for the entire study period and the two sub-periods 2006 – 2012 and 2013 – 2019. The parentheses contain the 95% confidence intervals.

|  | **Cold** | | **Heat** | |
| --- | --- | --- | --- | --- |
| **Outpatient Treatments** | **AF [%]** | **AN** | **AF [%]** | **AN** |
| Overall | 2.64 (0.27, 4.71) | 901 (92, 1609) | 1.06 (-0.92, 2.64) | 361 (-314, 902) |
| 2006 - 2012 | 3.36 (0.65, 5.72) | 430 (83, 732) | 0.85 (-0.92, 2.30) | 109 (-118, 295) |
| 2013 - 2019 | 2.21 (0.04, 4.14) | 472 (8, 884) | 1.18 (-0.91, 2.86) | 252 (-194, 611) |
| **Hospital Admissions** | **AF [%]** | **AN** | **AF [%]** | **AN** |
| Overall | 2.33 (0.45, 3.98) | 1002 (193, 1715) | -0.70 (-2.40, 0.81) | -302 (-1034, 348) |
| 2006 - 2012 | 2.66 (0.59, 4.48) | 544 (120, 917) | -0.66 (-2.15, 0.66) | -135 (-440, 134) |
| 2013 - 2019 | 2.02 (0.33, 3.54) | 459 (74, 803) | -0.74 (-2.62, 0.93) | -167 (-594, 210) |


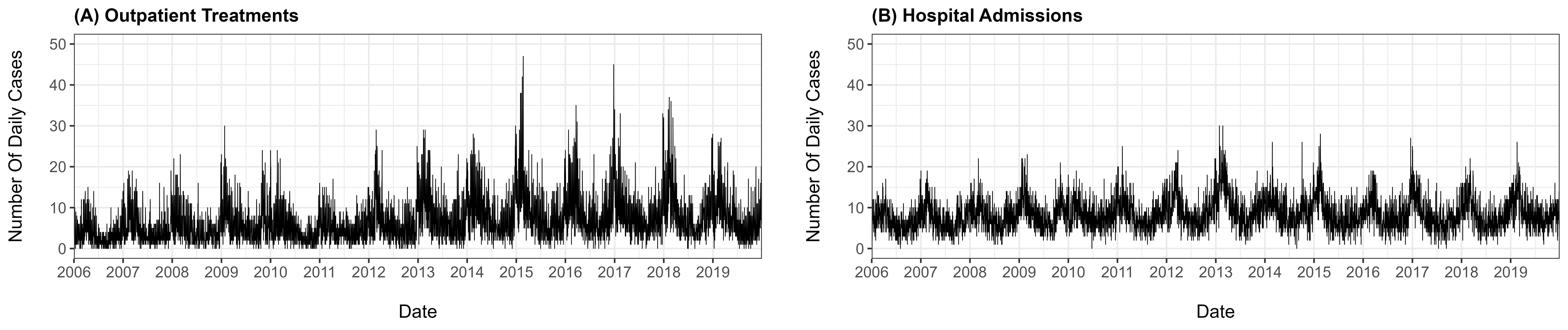


**Fig. S1** Daily number of emergency outpatient treatments (A) and hospital admissions (B) with a respiratory main diagnosis at the University Clinic of Augsburg. The y-axis shows the number of cases, the x-axis represents the study period between 2006 and 2019.


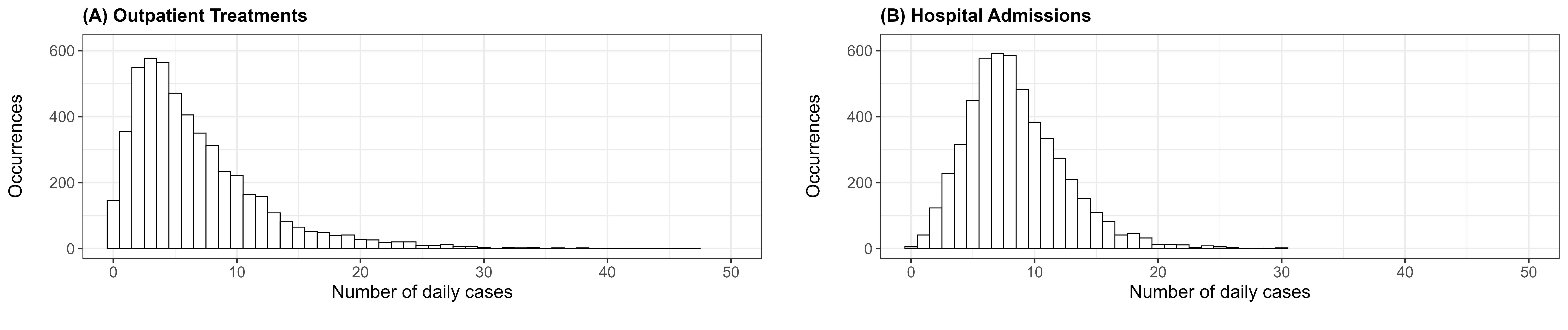


**Fig. S2** Distribution of the number of daily emergency outpatient treatments (A) and hospital admissions (B) with a respiratory main diagnosis in the study period between 2006 and 2019.


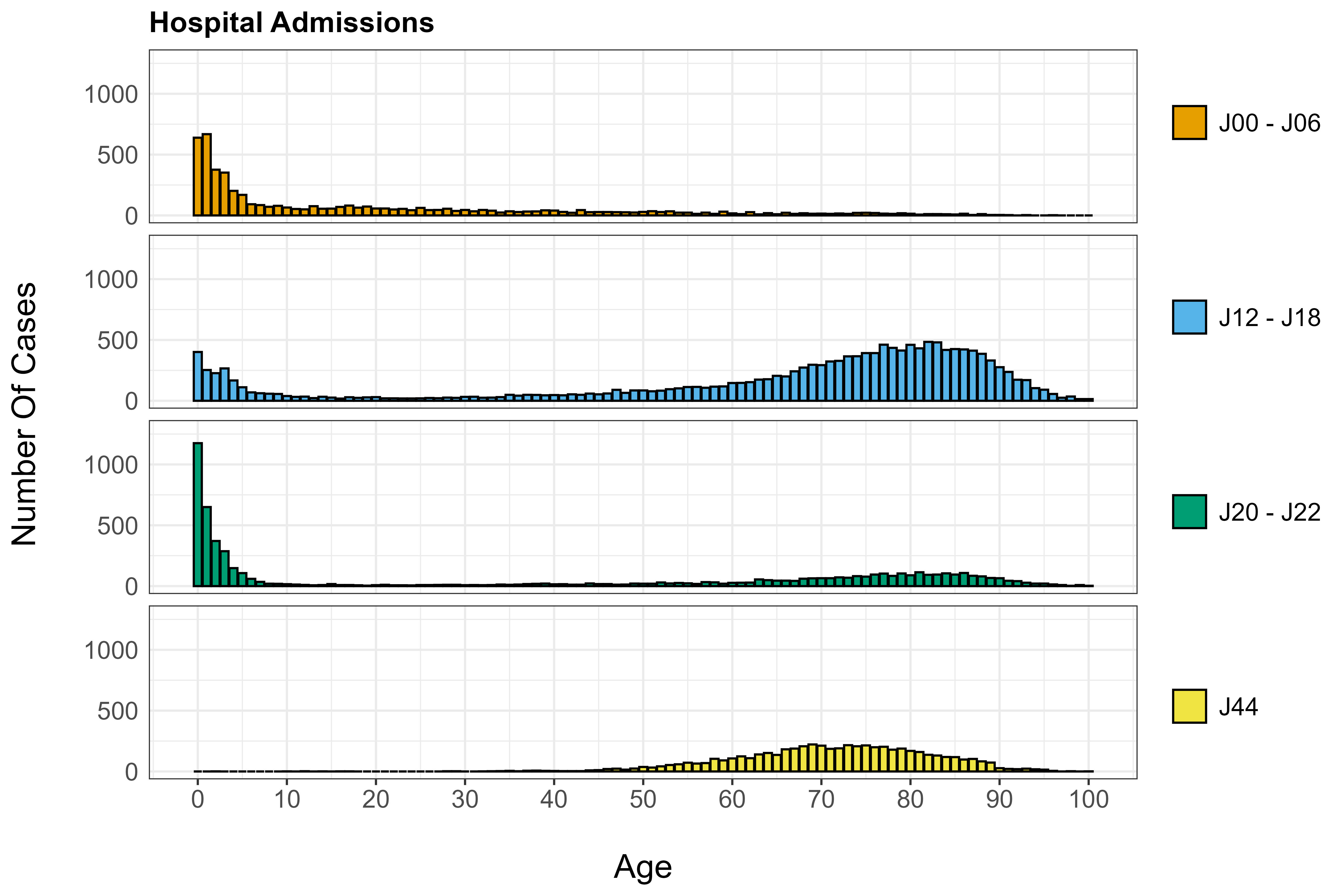


**Fig. S4** Distribution of hospital admissions for the most frequent ICD-10 respiratory sub-diagnoses across the patients’ age.


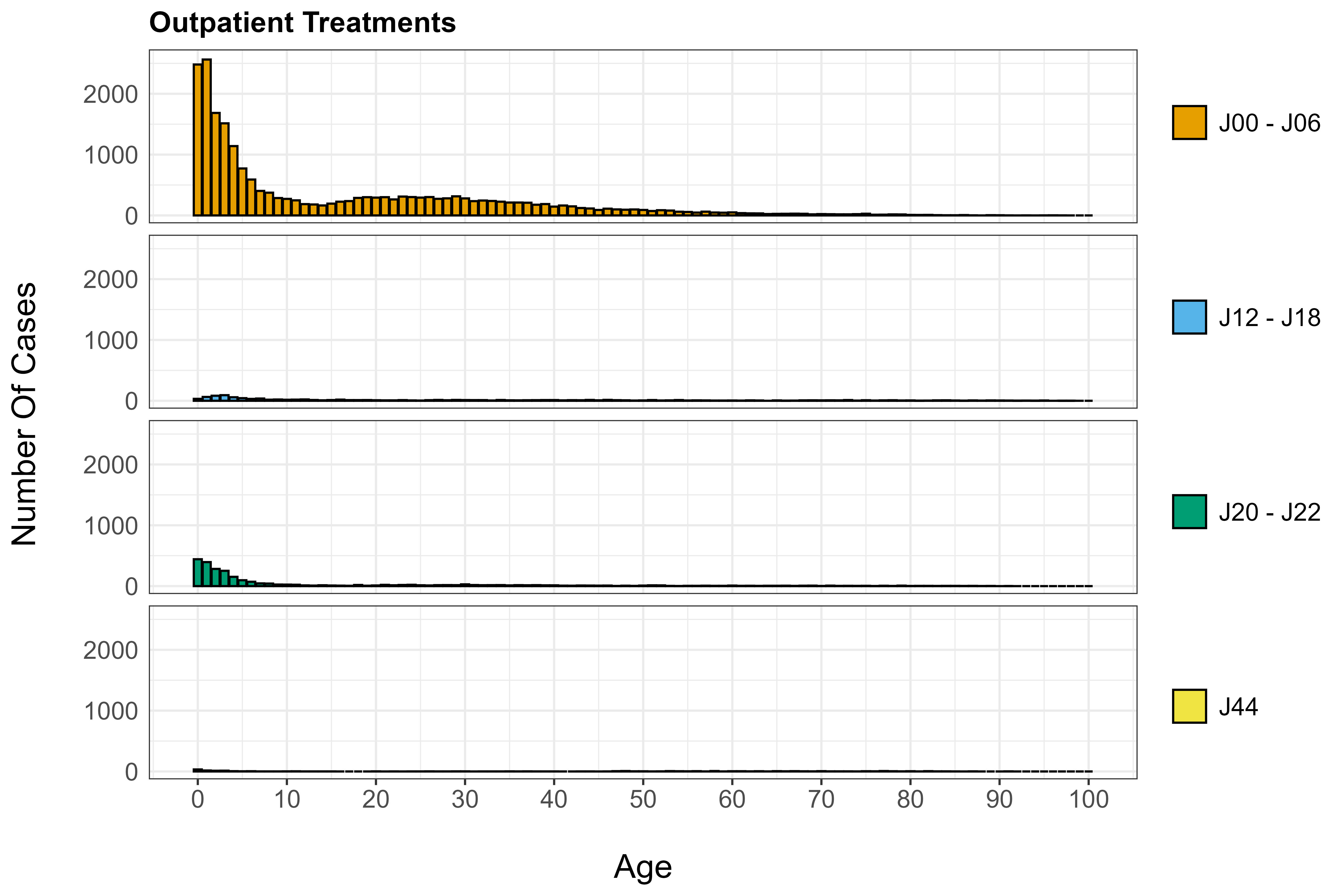


**Fig. S3** Distribution of outpatient treatments for the most frequent ICD-10 respiratory sub-diagnoses across the patients’ age.


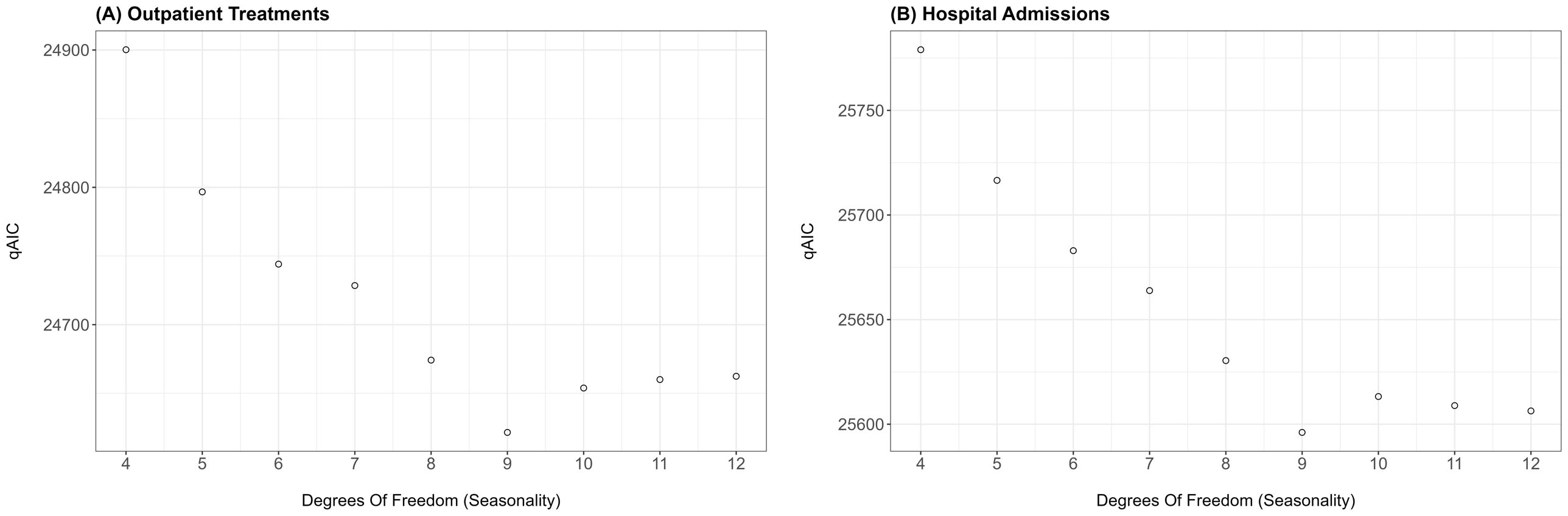


**Fig. S****5** QAIC values for the selection of the seasonal degrees of freedom per year for outpatient treatments (A) and hospital admissions (B).


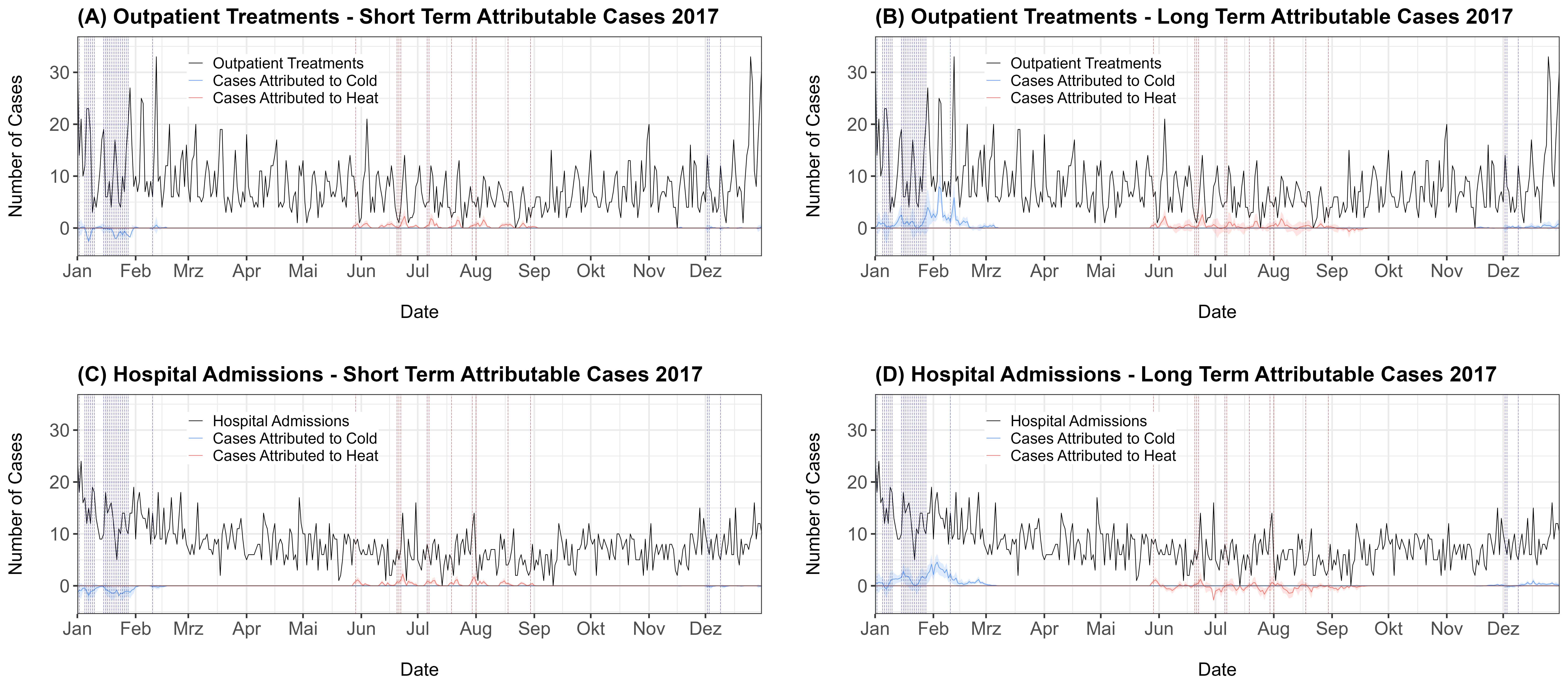


**Fig. S****6** Daily short-term (A, C) and long-term (B, D) attributable number of cases for outpatient treatments and hospital admissions due to cold and heat for the year 2017. The vertical dashed lines represent frost days (T_max_ < 0 °C, blue) and hot days (T_max_ >= 30 °C, red). Negative attributable case numbers are due to a protective effect relative to the median temperature.


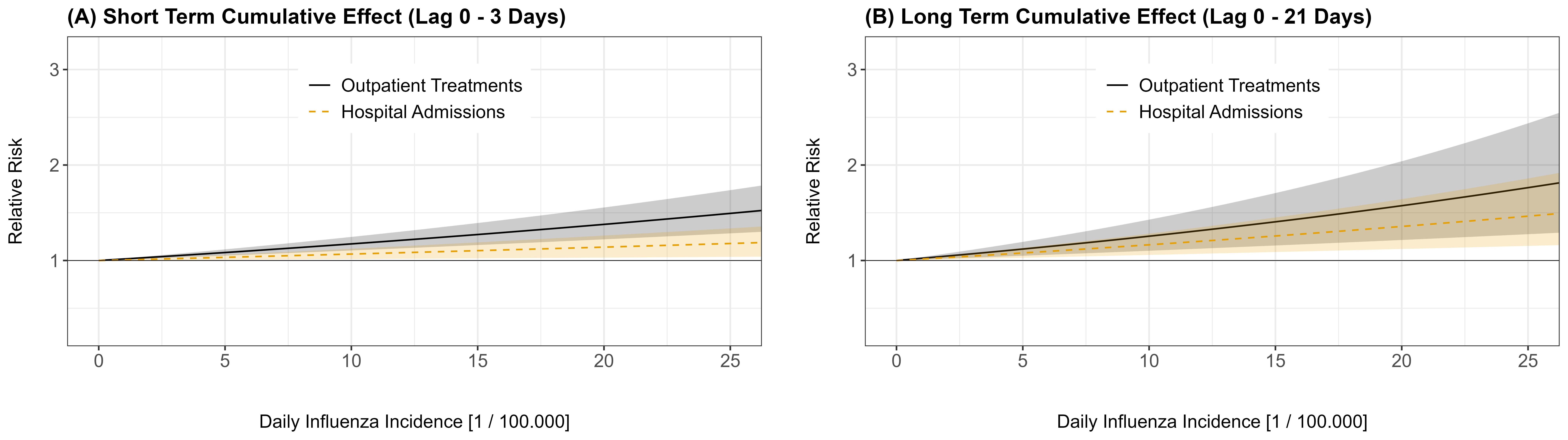


**Fig. S8** Short (A) and long (B) term cumulative effect of the influenza incidence on the daily number of outpatient treatments and hospital admissions reported as relative risk (RR). The shaded regions represent the 95% confidence intervals.


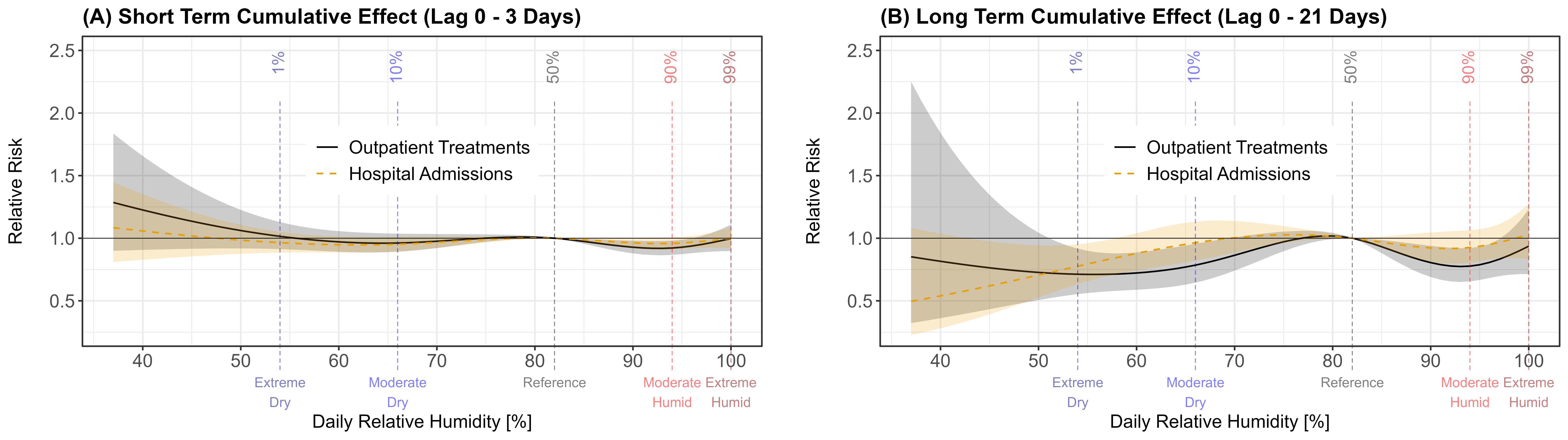


**Fig. S7** Short (A) and long (B) term cumulative effect of the relative humidity on the daily number of outpatient treatments and hospital admissions reported as relative risk (RR). The shaded regions represent the 95% confidence intervals.


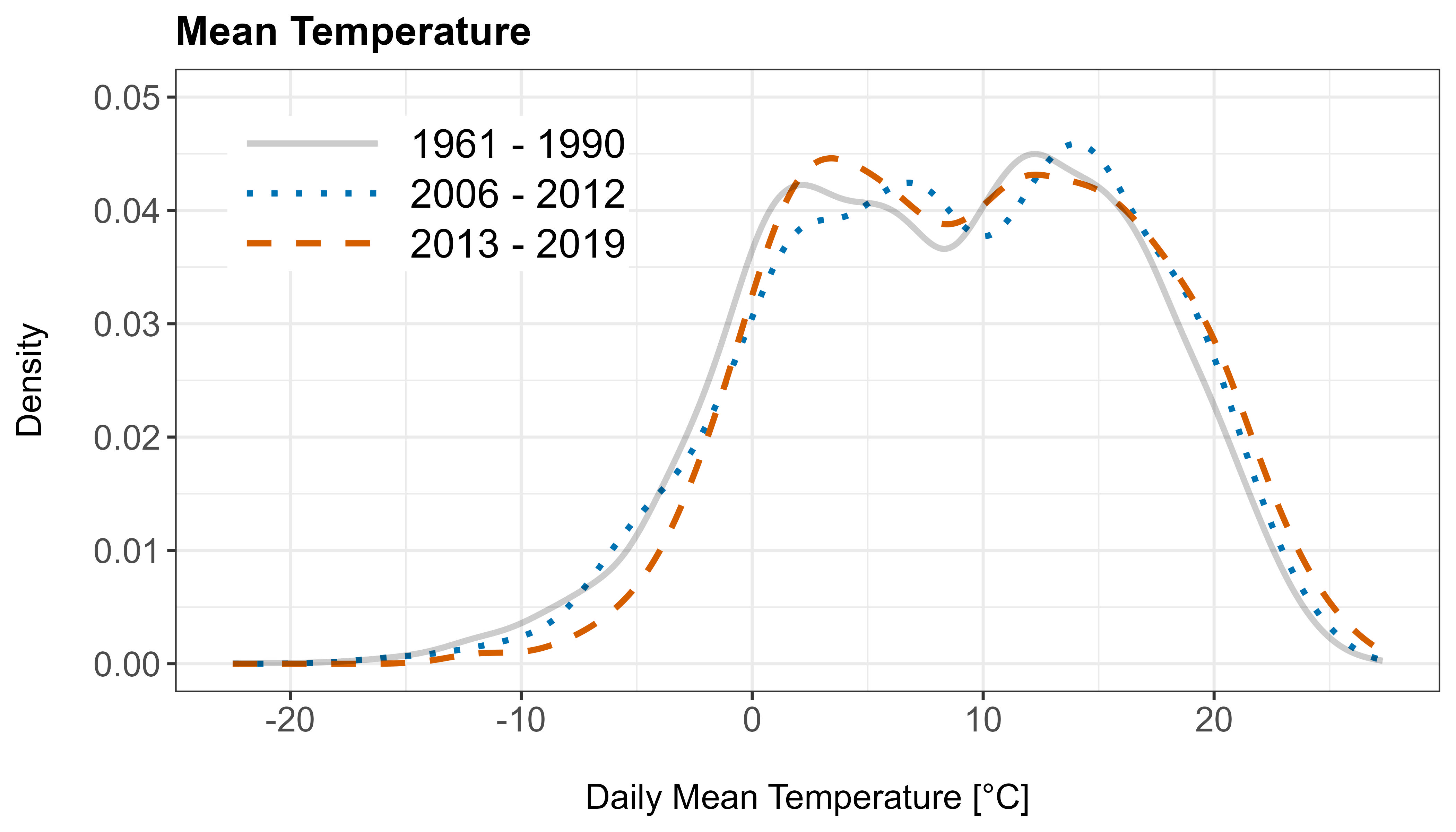


**Fig. S9** Density plot of the temperature distribution for the two sub-periods 2006 – 2012 and 2013 – 2019. The period between 1961 and 1990 is shown as a reference.


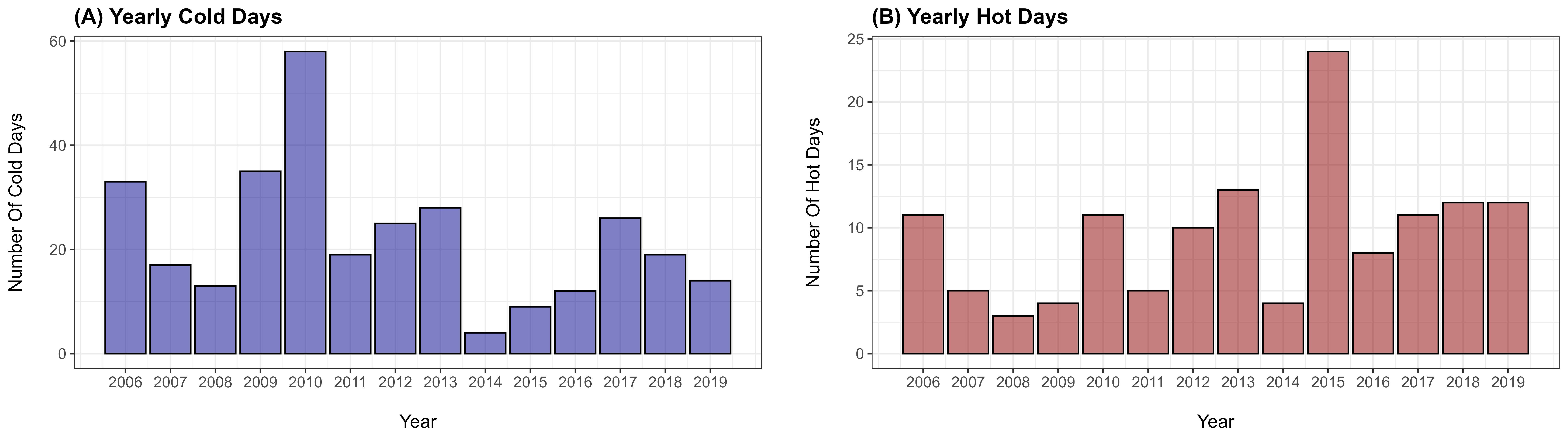


**Fig. S10** Bar diagram showing the number of yearly cold days (A) with maximum temperature (T_max_) measured below 0°C (T_max_ < 0 °C) and hot days (B) with T_max_ readings equal to or greater than 30 °C (T_max_ >= 30 °C).


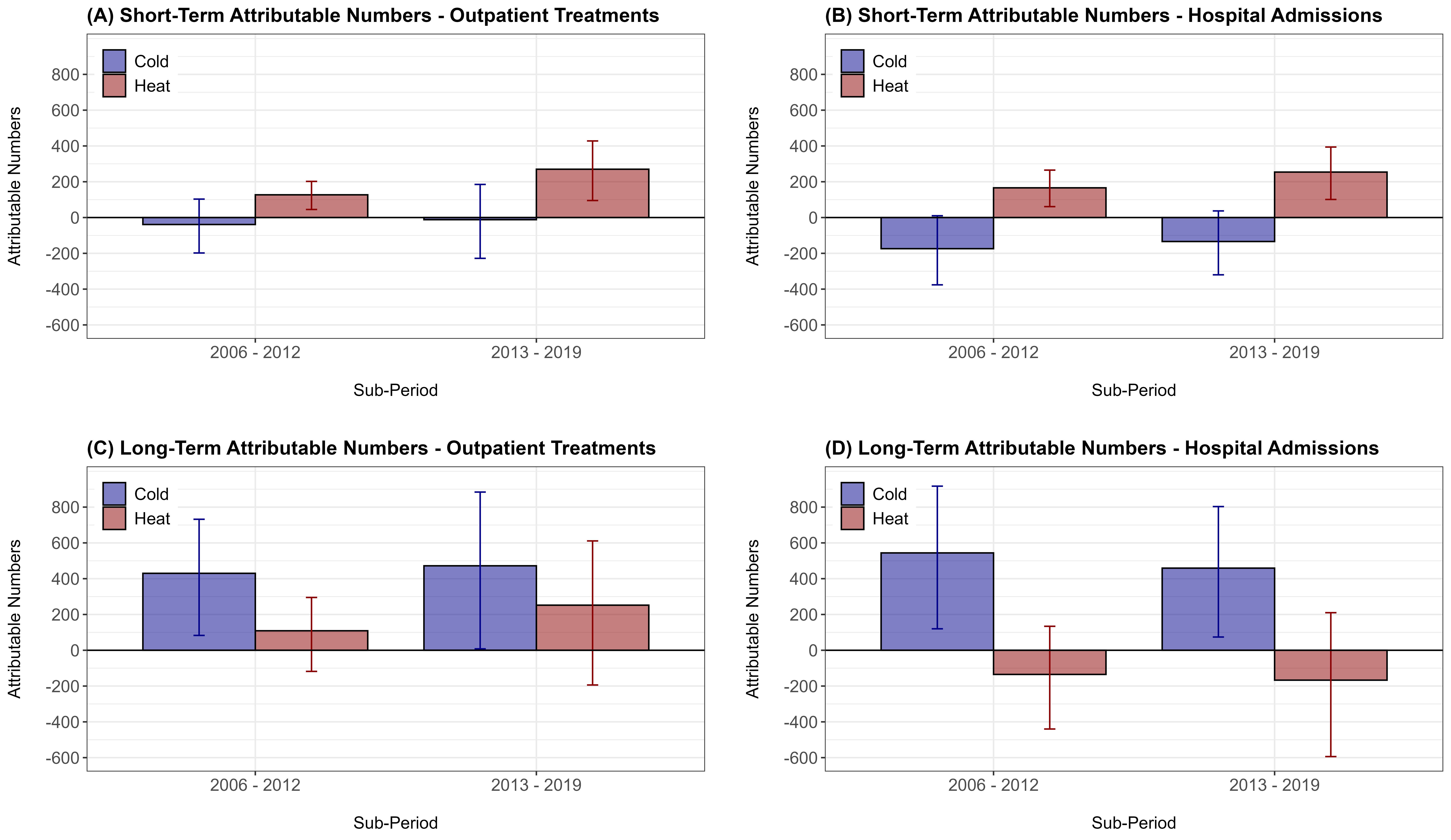


**Fig. S11** Bar plots showing the attributable numbers based on the short-term (top) and long-term (bottom) cumulative effect of cold (left) and heat (right) for the two sub-periods 2006 - 2012 and 2013 - 2019. The error bars represent 95% confidence intervals.


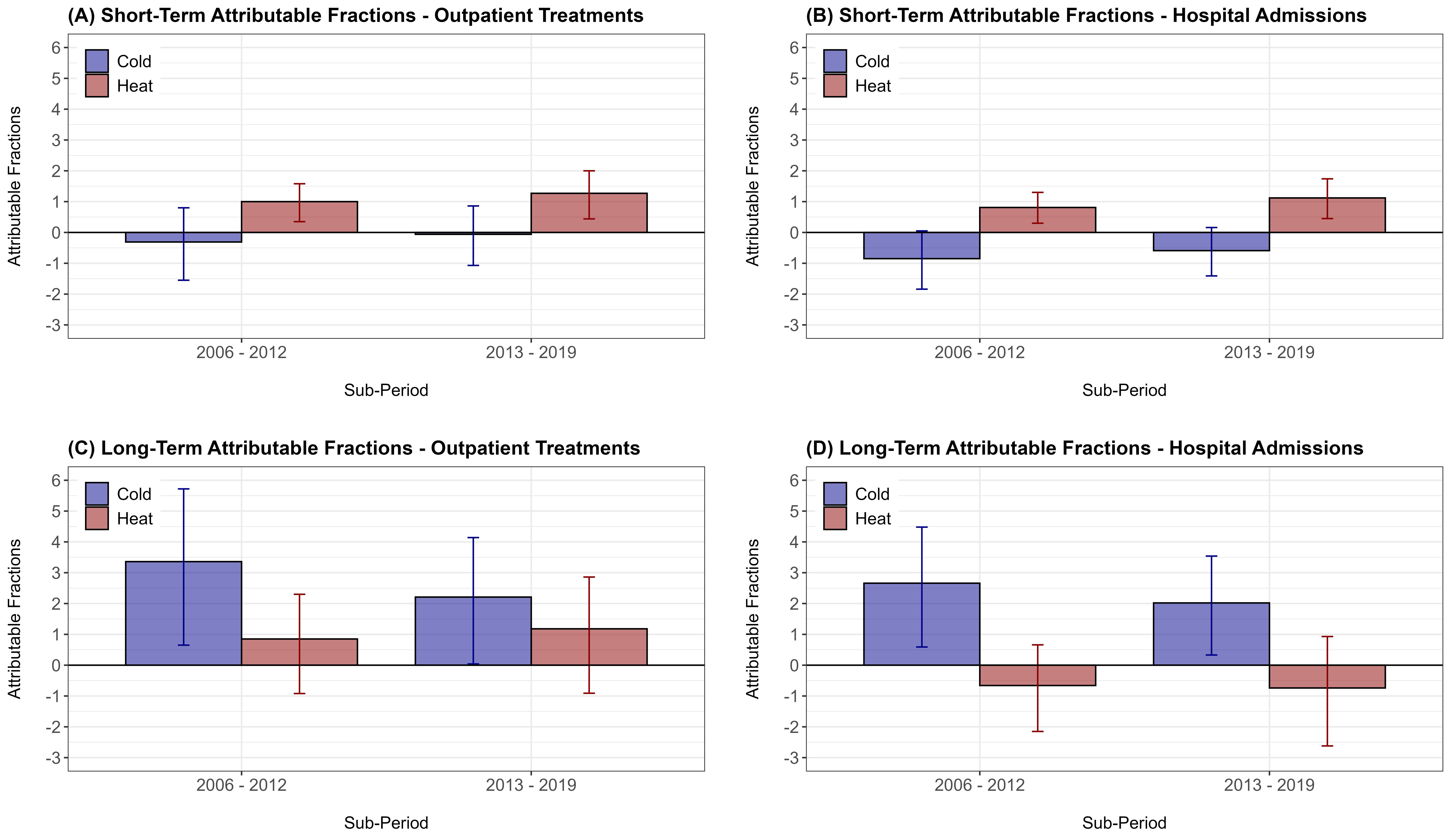


**Fig. S****12** Bar plots showing the attributable fractions based on the short-term (top) and long-term (bottom) cumulative effect of cold (left) and heat (right) for the two sub-periods 2006 - 2012 and 2013 - 2019. The error bars represent 95% confidence intervals.
